# Microbial bile salt hydrolases mediate the efficacy of faecal microbiota transplant in the treatment of recurrent *Clostridioides difficile* infection

**DOI:** 10.1136/gutjnl-2018-317842

**Published:** 2019-02-11

**Authors:** Benjamin H Mullish, Julie A K McDonald, Alexandros Pechlivanis, Jessica R Allegretti, Dina Kao, Grace F Barker, Diya Kapila, Elaine O Petrof, Susan A Joyce, Cormac G M Gahan, Izabela Glegola-Madejska, Horace R T Williams, Elaine Holmes, Thomas B Clarke, Mark R Thursz, Julian R Marchesi

**Affiliations:** 1 Division of Integrative Systems Medicine and Digestive Disease, Department of Surgery and Cancer, Faculty of Medicine, Imperial College London, London, UK; 2 Division of Gastroenterology, Hepatology and Endoscopy, Brigham and Women’s Hospital, Boston, Massachusetts, USA; 3 Harvard Medical School, Harvard University, Boston, Massachusetts, USA; 4 Division of Gastroenterology, Department of Medicine, University of Alberta, Edmonton, Alberta, Canada; 5 Division of Infectious Diseases/ GI Diseases Research Unit Wing, Department of Medicine, Kingston General Hospital, Queen’s University, Kingston, Ontario, Canada; 6 APC Microbiome Institute, University College Cork, Cork, Ireland; 7 School of Biochemistry and Cell Biology, University College Cork, Cork, Ireland; 8 School of Pharmacy, University College Cork, Cork, Ireland; 9 MRC Centre for Molecular Bacteriology and Infection, Imperial College London, London, UK; 10 School of Biosciences, Cardiff University, Cardiff, UK

**Keywords:** clostridioides difficile, bile acids, gut microbiota, metabonome, bile salt hydrolase

## Abstract

**Objective:**

Faecal microbiota transplant (FMT) effectively treats recurrent *Clostridioides difficile* infection (rCDI), but its mechanisms of action remain poorly defined. Certain bile acids affect *C. difficile* germination or vegetative growth. We hypothesised that loss of gut microbiota-derived bile salt hydrolases (BSHs) predisposes to CDI by perturbing gut bile metabolism, and that BSH restitution is a key mediator of FMT’s efficacy in treating the condition.

**Design:**

Using stool collected from patients and donors pre-FMT/post-FMT for rCDI, we performed 16S rRNA gene sequencing, ultra performance liquid chromatography mass spectrometry (UPLC-MS) bile acid profiling, BSH activity measurement, and qPCR of *bsh*/*bai*CD genes involved in bile metabolism. Human data were validated in *C. difficile* batch cultures and a C57BL/6 mouse model of rCDI.

**Results:**

From metataxonomics, pre-FMT stool demonstrated a reduced proportion of BSH-producing bacterial species compared with donors/post-FMT. Pre-FMT stool was enriched in taurocholic acid (TCA, a potent *C. difficile* germinant); TCA levels negatively correlated with key bacterial genera containing BSH-producing organisms. Post-FMT samples demonstrated recovered BSH activity and *bsh*/*bai*CD gene copy number compared with pretreatment (p<0.05). In batch cultures, supernatant from engineered *bsh*-expressing *E. coli* and naturally BSH-producing organisms (*Bacteroides ovatus, Collinsella aerofaciens, Bacteroides vulgatus* and *Blautia obeum*) reduced TCA-mediated *C. difficile* germination relative to culture supernatant of wild-type (BSH-negative) *E. coli. C. difficile* total viable counts were ~70% reduced in an rCDI mouse model after administration of *E. coli* expressing highly active BSH relative to mice administered BSH-negative *E. coli* (p<0.05).

**Conclusion:**

Restoration of gut BSH functionality contributes to the efficacy of FMT in treating rCDI.

**Significance of this study**

**What is already known on this subject?**

- Faecal microbiota transplant (FMT) effectively treats recurrent *Clostridioides difficile* infection (rCDI), but has drawbacks associated with its use. Furthermore, its mechanisms of action remain poorly defined.
- There is an association between rCDI and altered gut bile acid profiles, but the significance of this remains unclear.

**What are the new findings?**

- Patients successfully treated for rCDI by FMT showed a rapid and sustained enrichment in gut microbiota members which produce a bile-metabolising enzyme, bile salt hydrolase (BSH).
- Relative abundance of gut BSH-producing organisms correlates negatively with stool levels of taurocholic acid (TCA), a potent trigger for *C. difficile* germination. TCA is hydrolysed by BSH to cholic acid; this is subsequently metabolised into deoxycholic acid, which potently inhibits the growth of *C. difficile*.
- In batch cultures, BSH-producing microorganisms completely reversed TCA-mediated germination of *C. difficile.*
- BSH-producing microbes significantly reduced faecal counts of *C. difficile* after administration to an rCDI mouse model compared with administration of a BSH-negative microbes.

**How might it impact on clinical practice in the foreseeable future?**

- Restored gut BSH functionality plays a central role in the efficacy of FMT in treating CDI.
- Targeted restoration of this function within the gut (eg, BSH-producing microorganisms, purified BSH enzyme) represents a novel therapeutic approach to treating rCDI which avoids the limitations associated with FMT.
- BSH supplementation merits further evaluation for its potential role as a therapy for human patients with rCDI.

## Introduction

*Clostridioides difficile* (previously named *Clostridium difficile* 1) infection (CDI) is the major global cause of nosocomial GI infection, with incidence rates increasing worldwide.^2 3^ One therapeutic strategy for recurrent CDI (rCDI) that has come to prominence is faecal microbiota transplant (FMT). FMT has been demonstrated to be a highly effective therapy for rCDI, with cure rates of >80% in randomised trials,^4^ and up to >90% in case series where it was administered colonoscopically.^5^ However, FMT is not without drawbacks, including its unpalatability, the theoretical risk of infection transmission and its regulatory complexity.^6^

Understanding the mechanisms underlying the efficacy of FMT in treating CDI may allow formulation of novel, more targeted, anti-CDI therapeutics. In a pilot study, sterile faecal filtrate was shown to be an effective treatment for rCDI in five patients,^7^ consistent with bacterially derived proteins, gut metabolites, bacteriophages or other filtrate components mediating the efficacy of FMT in treating this condition, as opposed to intact microorganisms. While the possible contribution of such mediators to FMT are starting to be elucidated,^8–10^ they remain incompletely defined.

One particular area of interest concerns the interaction between the gut microbiota and host bile acid metabolism in rCDI. In vitro, certain bile acids differentially affect the ability of *C. difficile* to undergo germination and vegetative growth. In particular, the conjugated primary bile acid taurocholic acid (TCA) is a potent trigger of *C. difficile* germination (with glycine as co-germinant),^11^ while the secondary bile acid deoxycholic acid (DCA) markedly inhibits vegetative growth.^11 12^ The transition from conjugated primary bile acids to secondary bile acids in vivo principally involves two enzymatic steps, with both enzymes produced by microbes but not mammals (see online supplementary figure 1). The first step is undertaken by bile salt hydrolases (BSHs), which deconjugate the taurine and glycine groups via a hydrolysis reaction, and consequently reform the unconjugated primary bile acids cholate and chenodeoxycholate. BSHs are widely distributed throughout most major bacterial divisions and archaea species of the gut microbiota, and at least eight different *bsh* genes exist^13^ (see online supplementary figure 2). The second enzymatic step is 7-α-dehydroxylation, whereby unconjugated primary bile acids are converted to secondary bile acids, including deoxycholate and lithocholate.

10.1136/gutjnl-2018-317842.supp1 Supplementary data

At present, there are only limited data exploring the possible contribution of BSHs to CDI vulnerability,^14^ and none investigating the effect of FMT on BSH functionality. Given the key contribution of BSH’s substrate, TCA, to the germination of *C. difficile,* this is a clear potential mechanistic explanation for the efficacy of FMT. As such, using a work flow including human samples, batch cultures and mouse models, we investigated the hypothesis that patients with rCDI are deficient in gut microbiota members which produce BSH, with the consequent enrichment in TCA (promoting *C. difficile* germination) and loss of DCA (permitting vegetative growth) contributing to ongoing disease. We further hypothesised that successful FMT recolonises the gut microbiota with BSH-producing organisms, contributes to the restoration of the normal bile acid *milieu* of the gut, and consequently removes key triggers for *C. difficile* germination and vegetative growth.

## Materials and methods

### Study participants and FMT protocols

For the main human data set, stool samples were collected from participants with rCDI (26 participants; samples collected pre- FMT and at 8–12 weeks post-FMT) and their FMT donors (17 participants). rCDI was diagnosed on a combination of clinical and laboratory criteria (see online supplementary methods 1.1). Patient characteristics are described in online supplementary table 1; none of the included patients had IBD. For validation of initial findings, stool samples were also analysed for patients with rCDI from a Canadian randomised controlled trial investigating capsulised versus colonoscopic FMT as rCDI treatment (18 participants; samples collected pre-FMT and at 1, 4 and 12 weeks post-FMT), together with donors (five participants).^15^ FMT slurry was also collected from these donors.

Donor inclusion/exclusion criteria, screening and testing followed previously described recommendations.^16^ FMT protocols used are detailed in online supplementary methods 1.1.

### DNA extraction and 16S rRNA gene sequencing

DNA was extracted from 250 mg of stool using a previously described protocol.^17^ 16S rRNA gene qPCR data were used to determine total bacterial biomass within each sample (see online supplementary methods 1.2). Sample libraries were prepared following Illumina’s 16S Metagenomic Sequencing Library Preparation Protocol^18^ with several modifications.^17^ The V1-V2 regions of the 16S rRNA gene were amplified using previously reported primers.^17^ The methodology for metataxonomic analysis is described in online supplementary methods 1.3. We also predicted the bile metabolising ability of microbial communities using a metagenomic inferential tool, Piphillin^19^ (see online supplementary methods 1.4).

### UPLC-MS profiling of faecal bile acids

The protocol used for initial sample processing^17^ and for analysis^20^ was as previously described. Methodology for preprocessing and analysis of ultra performance liquid chromatography mass spectrometry (UPLC-MS) bile acid data is described in online supplementary methods 1.5.1. Integration of metataxonomic and UPLC-MS bile profiling data is described in online supplementary methods 1.6

### Abundance and activity of bile metabolising enzymes

qPCR was performed using extracted DNA to quantify gene abundance for (1) specified groups of *bsh* (using degenerate primer sets previously reported)^17^ and (2) *bai*CD (using previously described primers^21^) (see online supplementary methods 1.7). The BSH activity assay was an adaptation of the conventional precipitation-based assay,^22–24^ using a previously described technique.^17^

### *C. difficile* germination batch cultures

These were performed via adaptation of a previously described protocol.^11^ *C. difficile* spore preparation and enumeration is described in online supplementary methods 1.8. A range of different bacterial species established to produce BSH from different BSH groups (and *Clostridium scindens,* as a known 7-α-dehydroxylase-producer) were incubated in sBHI (brain heart infusion broth (Sigma-Aldrich), with 5 mg/mL yeast extract (Sigma-Aldrich), and 0.1% w/v L-cysteine (Sigma-Aldrich)), with or without 1% w/v TCA added (see online supplementary methods 1.7). This list also included wild-type *E. coli* MG1655 (which does not contain *bsh* genes within its genome), along with two forms of *E. coli* MG1655 into which *bsh* genes had been cloned using pBKminiTn*7*GM2 under the control of the P44 promotor^25^ (see online supplementary methods 1.7). *C. difficile* spores from three different ribotypes (a non-toxigenic ribotype, 010 (strain DS1684), and two toxigenic ribotypes, 012 (strain CD630) and 027 (strain R20291)) were resuspended in supernatant in triplicate, and an OD_600_ reading taken on a microplate reader at time zero (adjusted to OD_600_ of ~0.1 with supernatant/sBHI mix), and again after overnight incubation. An increased OD_600_ reading after overnight incubation was interpreted as indicating that spores had undergone germination and had grown as vegetative cells.^11^ Additionally, UPLC-MS was performed on batch culture supernatants to establish bile acid profiles (see online supplementary methods 1.5.2), and BSH activity assays were performed on spent supernatant from selected batch cultures.

### Recurrent *C. difficile* mouse model

Wild-type C57BL/6 mice (8–10 weeks old; female) were purchased from Envigo (Huntingdon, UK) and acclimatised for 1 week before use. Mice were housed five per cage in individually ventilated cages with autoclaved food (RM1, Special Diet Services, Essex, UK), bedding (Aspen chip two bedding, Northeastern Products Corporation (NEPCO), Warrensburg, New York) and water (provided ad libitum). Mice were subjected to a 12 hours light and 12 hours dark cycle at 20°C–22°C.

**Figure 1:**
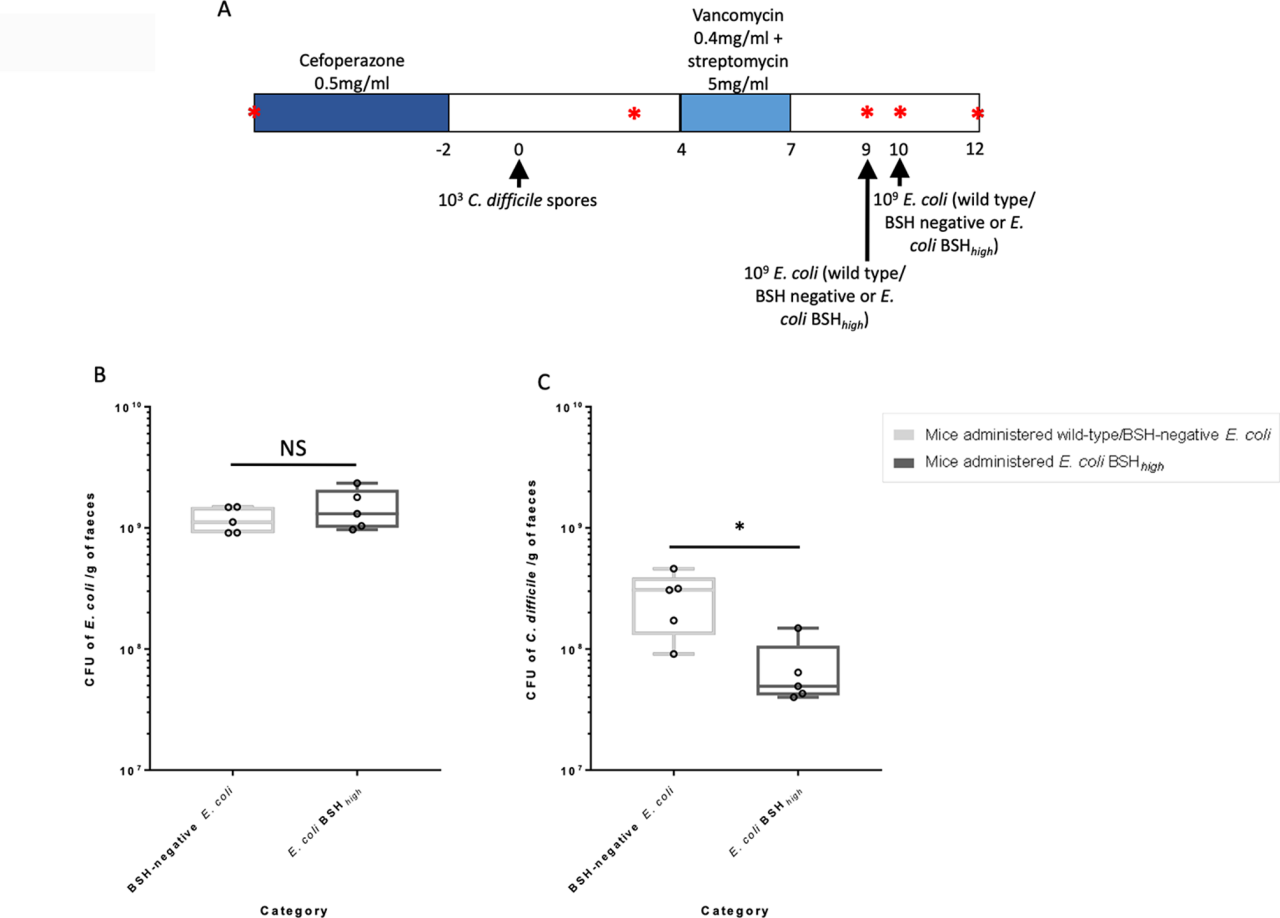
Impact of BSH on a mouse model of rCDI. A mouse model of rCDI/FMT was used that was adapted from a previously described model.^26^ (A) Protocol for mouse model (*: stool samples taken for *C. difficile* and/or *E. coli* counts); (B) Counts of administered *E. coli* on day 12; (C) Total vegetative counts of *C. difficile* on day 12. CFU counts as established from plate counts of serial dilutions of faecal supernatant (*, p<0.05, Mann-Whitney U test). BSH, bile salt hydrolases; CFU, colony-forming unit; FMT, faecal microbiota transplant; rCDI, recurrent *C. difficile* infection.

We adapted a previously published mouse model of rCDI/FMT^26^ (figure 1A). Mice were initially given cefoperazone 0.5 mg/mL (Melford, Ipswich, UK) in their drinking water for 5 days (day −7 to day −2), followed by challenge with 10^3^ *C. difficile* spores by oral gavage on day 0. Mice were then treated with vancomycin 0.4 mg/mL together with streptomycin 5 mg/mL (both Melford, Ipswich, UK) in their drinking water for 3 days (days 4–7), followed by autoclaved antibiotic-free water for the remainder of the experiment. On both days 9 and 10, mice were fed by oral gavage either with ~10^9^ colony-forming units (CFUs) of wild-type *E. coli* (n=5) or *E. coli* BSH*_high_* (n=5) (see online supplementary methods 1.7). Serial faecal samples were collected and *C. difficile* total viable counts (TVCs) quantified until *E. coli* colonisation began to decline (see online supplementary methods 1.9). Administered *E. coli* were quantified by plating on MacConkey agar plates supplemented with rifampicin 50 µg/mL (Melford, Ipswich, UK). Mice were not fasted before oral gavages and all interventions were performed during the light cycle.

### Statistical analysis

This is summarised in online supplementary methods 1.10.

## Results

### Successful FMT for rCDI is associated with restoration of BSH-producing gut microbial community members from all BSH groups

We used microbial sequencing to evaluate whether FMT for rCDI was associated within an increase in BSH-producing microbes within the faecal microbiota. Further analysis of metataxonomic data is provided in online supplementary results, figures 3–5 and table 2.

**Figure 2:**
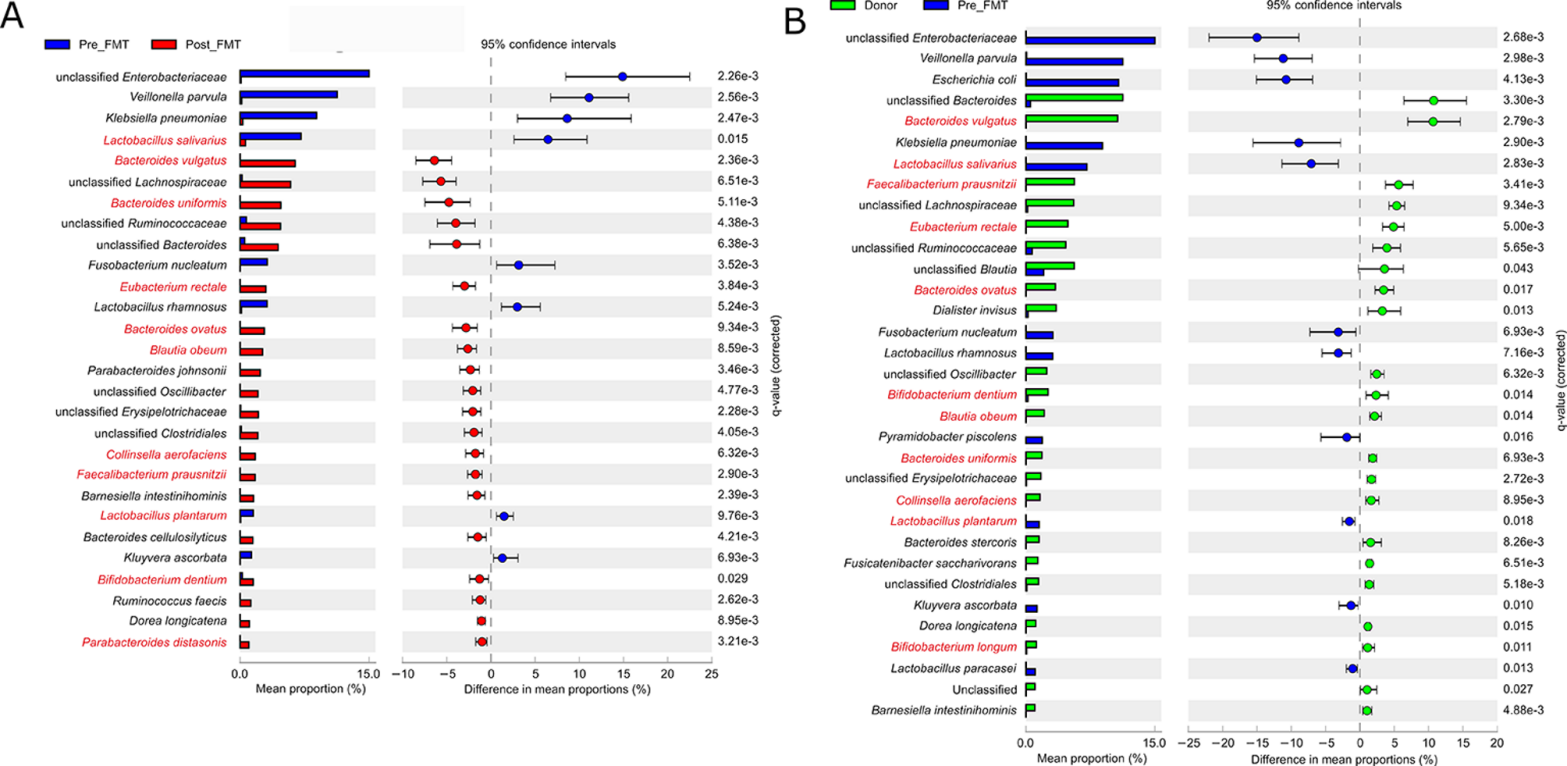
Species differences in 16S rRNA gene sequencing data in patients with rCDI compared with donor or post-FMT. Extended error bar plots, with bacterial species changing significantly measured by White’s non-parametric test with Benjamini-Hochberg correction, using threshold of differences between mean proportions >1%. (A) Donor versus pre-FMT; (B) Pre-FMT versus post-FMT. Names in red: known BSH-producing bacteria. BSH, bile salt hydrolases; FMT, faecal microbiota transplant; rCDI, recurrent *C. difficile* infection.

Bacterial species enriched in the faecal microbiota of healthy donors compared with pre-FMT samples were characterised by a range of BSH-producing organisms, including members of group 1 (*Bacteroides ovatus, q=*0.017; *Bacteroides uniformis, q*=0.007), group 2 (*Bifidobacterium dentium, q*=0.014; *Collinsella aerofaciens, q*=0.009; *Bifidobacterium longum, q*=0.011) and group 3 (*Bacteroides vulgatus, q*=0.003; *Faecalibacterium prausnitzii, q=*0.003; *Eubacterium rectale, q*=0.005; *Blautia obeum, q*=0.014) (figure 2A). Similarly, bacterial species enriched in the post-FMT faecal microbiota compared with those pre-FMT also included members of all BSH groups, including group 1 (*Bacteroides uniformis, q=*0.005; *Bacteroides ovatus, q=*0.009; *Parabacteroides distasonis, q*=0.003), group 2 (*Collinsella aerofaciens, q*=0.006; *Bifidobacterium dentium, q*=0.029) and group 3 (*Bacteroides vulgatus, q*=0.002; *Eubacterium rectale, q*=0.004; *Blautia obeum, q*=0.009; *Faecalibacterium prausnitzii, q*=0.003) (figure 2B).

Using Piphillin to infer metagenomic content from metataxonomic data, there were a significantly reduced proportion of sequences predicted to represent both BSH (see online supplementary figure 6A) and 7-α-dehydroxylase (see online supplementary figure 6B) in pre-FMT samples compared with donors, but a significant increase in both in post-FMT samples compared with pre-FMT (*q*<0.01, White’s non-parametric test with Benjamini-Hochberg false discovery rate (FDR)).

### Successful FMT for rCDI is associated with restoration of normal gut bile acid profiling, including a sustained reduction in TCA

We performed faecal bile acid profiling to assess the effect of FMT for rCDI on key bile acids known to affect the ability of *C. difficile* to undergo germination or vegetative growth.

**Figure 3:**
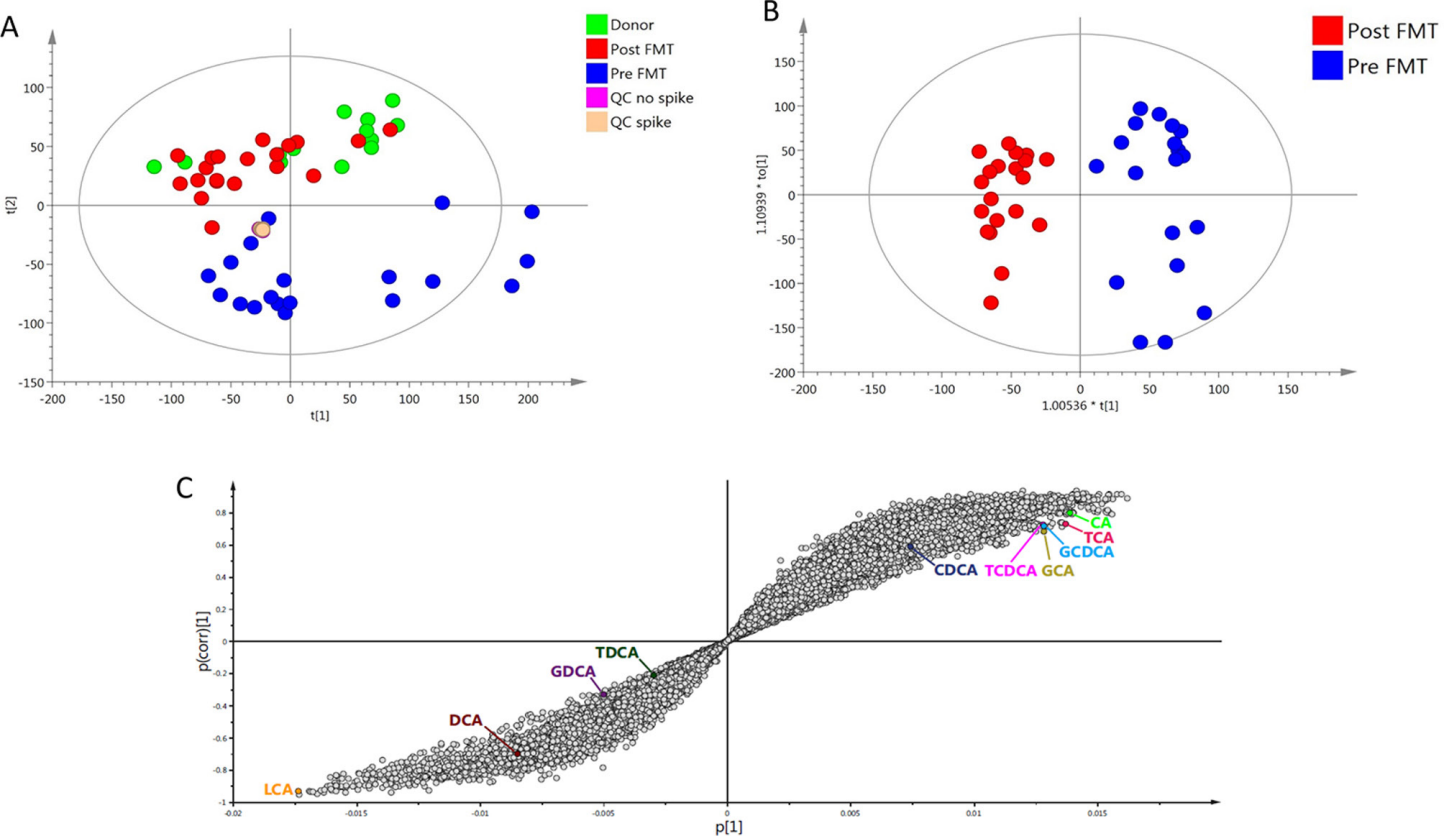
Effect of FMT for rCDI on stool bile acid profiles. Assessed via multivariate analysis of UPLC-MS bile acid profiling data. (A) PCA scores plot; (B) OPLS-DA scores plot, comparing pre-FMT and post-FMT samples; (C) OPLS-DA S-plot of pre-FMT versus post-FMT data. FMT, faecal microbiota transplant; OPLS-DA, orthogonal projections to latent structures discriminant analysis; PCA, principal component analysis; QC, quality controls; rCDI, recurrent *C. difficile* infection; UPLS-MS, ultra performance liquid chromatography mass spectrometry.

On multivariate analysis of UPLC-MS bile acid profiling data, unsupervised principal component analysis demonstrated clustering of donor and post-FMT samples, but clear separation of both groups from pre-FMT samples (figure 3A). Supervised analysis was performed with orthogonal projections to latent structures discriminant analysis (OPLS-DA) to analyse the features responsible for discrimination between donor and pre-FMT groups (see online supplementary figure 7A), and between pre-FMT and post-FMT groups (figure 3B). Discriminatory feature identification was performed from OPLS-DA model data via S-plot, with pre-FMT samples showing an enrichment in primary bile acids (including both conjugated and unconjugated forms) and loss of secondary bile acids as compared with post-FMT and healthy donor samples (figure 3C, see online supplementary figure 7B). OPLS-DA model validation was performed using CV analysis of variance (see online supplementary table 3).

Univariate analysis supported these findings (see online supplementary figure 8). Pre-FMT samples demonstrated enrichment in TCA and loss of DCA compared with healthy donor samples (p<0.01, Mann-Whitney U test), while post-FMT samples were characterised by restoration of both bile acids back to levels comparable to donors (p<0.001, Wilcoxon signed rank-sum test).

### Integration of metataxonomic and bile acid profiling data

**Figure 4:**
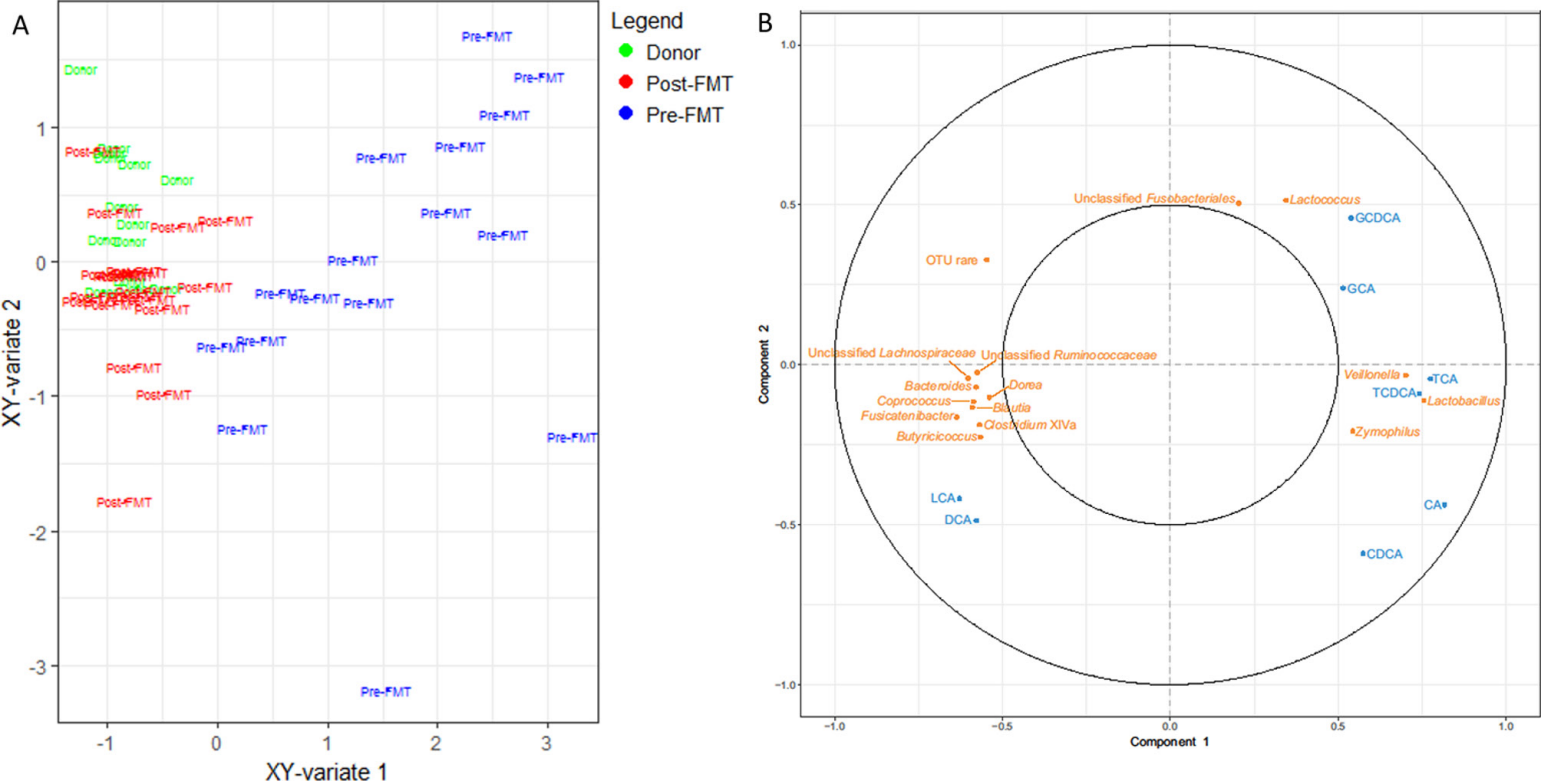
Regularised CCA (rCCA) model correlating 16S rRNA gene sequencing data (genus level) and bile acid data. (A) Unit representation plot for the two canonical variables (metataxonomics and stool bile acids); (B) Correlation circle plot between pre-FMT and post-FMT samples. Bile acids are shown in blue and bacterial genera are shown in orange. FMT, faecal microbiota transplant.

rCCA modelling was used to integrate metataxonomic and bile acid profiling data. The unit representation plot demonstrated marked separation of pre-FMT and post-FMT samples, but considerable overlap between donor and post-FMT samples (figure 4A). A correlation circle plot demonstrated negative correlations between levels of TCA and the abundance of the bacterial genera *Bacteroides* and *Blautia,* both known to include representative BSH-producing organisms that were significantly increased after FMT in our metaxonomic data (figure 4B). In addition, there was positive correlation between the genus *Clostridium* cluster XIVa (known to contain 7-α-dehydroxylase producing organisms^27 28^) and the secondary bile acids DCA and lithocholic acid (figure 4B).

### Gut *bsh* gene copy number and BSH enzyme activity is restored by successful FMT for rCDI

Having established an association between restoration of BSH-producing organisms into the gut microbiota post-FMT and recovery of premorbid stool bile acid profiles, we investigated the effect of FMT for rCDI on BSH expression and activity.

**Figure 5:**
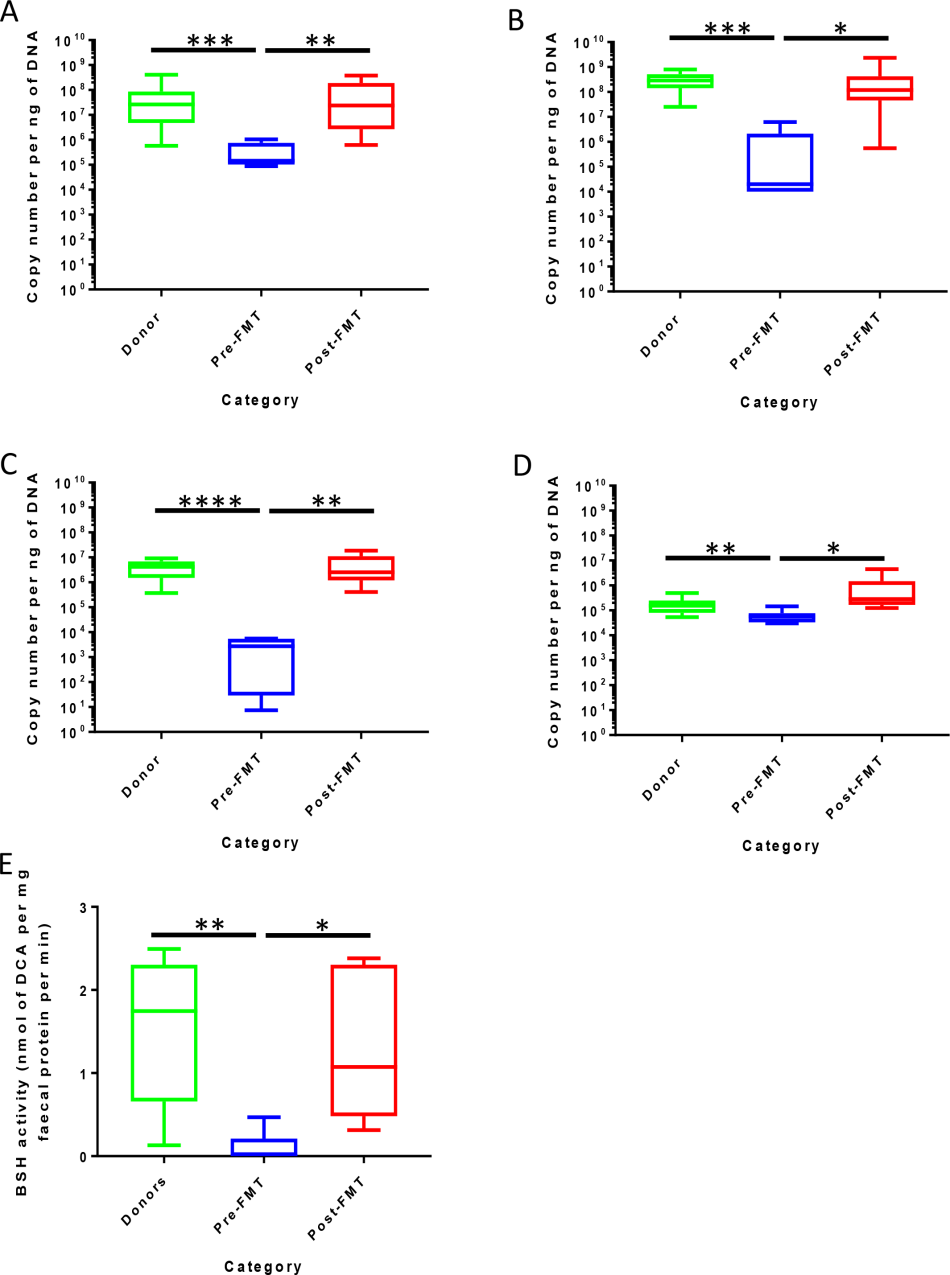
Effect of FMT on bile-metabolising enzyme gene copy number and BSH enzyme activity. (A) *bsh* group 1A; (B) *bsh* group 1B; (C) *bsh* group 3C; (D) *bai*CD operon of 7-α-dehydroxylase; (E) BSH enzyme activity within faecal supernatant (*, p<0.05; **, p<0.01; ***; p<0.001; ****, p<0.0001; Mann-Whitney U test for donors versus pre-FMT, Wilcoxon signed rank-sum test for pre-FMT vs post-FMT). BSH, bile salt hydrolases; FMT, faecal microbiota transplant.

*bsh* gene copy number was significantly reduced in pre-FMT samples compared with healthy donors across a range of *bsh* gene groups (p<0.001, Mann-Whitney U test, figure 5A–C). Successful FMT was associated with significant enrichment in copy number of all *bsh* gene groups assayed (p<0.05, Wilcoxon signed rank-sum test, figure 5A–C) to levels similar to that of healthy donors. Similarly, copy numbers of the *bai*CD operon (encoding an enzyme that contributes to 7-α-dehydroxylation^29^) were significantly lower in pre-FMT samples compared with those of healthy donors (p<0.01), but were also significantly enriched post-FMT (p<0.05, figure 5D). Gene copy number for *bai*CD in donors and post-FMT was noted to be markedly lower than for each *bsh* gene assayed for the same participants. Stool BSH enzyme activity showed the same pattern as for *bsh* gene copy number (figure 5E).

**Figure 6:**
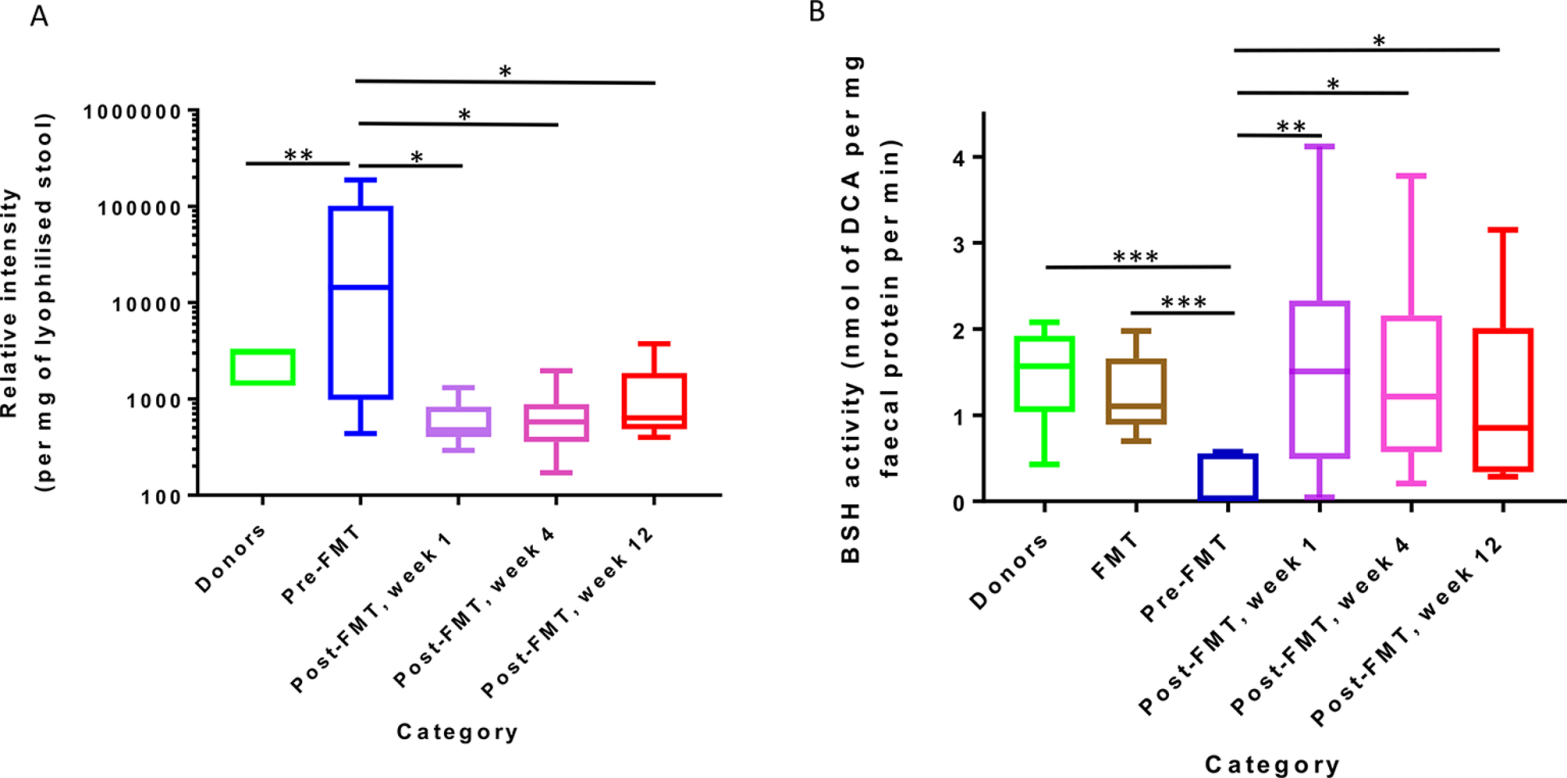
Dynamics of changes in taurocholic acid and BSH activity after FMT for rCDI. Assessed via analysis of taurocholate levels in stool ((A) as assessed by UPLC-MS) and BSH activity in stool and FMT slurry ((B) assessed via precipitation assay). Samples were collected from patients (and their matched donors, along with FMT slurry itself) in a randomised trial of colonoscopy versus capsule FMT as treatment for rCDI^15^ (*, p<0.05; **, p<0.01; ***, p<0.001; Mann-Whitney U test for donors/FMT slurry versus pre-FMT, Friedman test with Benjamini-Hochberg FDR for pre-FMT vs post-FMT). BSH, bile salt hydrolases; DCA, deoxycholic acid; FMT, faecal microbiota transplant; rCDI, recurrent *C. difficile* infection; UPLC-MS, ultra performance liquid chromatography mass spectrometry.

To further explore the timescale of gut bile acid/BSH changes post-FMT, we analysed serial stool samples collected from patients in a randomised trial of colonoscopy versus capsule FMT as treatment for rCDI,^15^ together with donor samples and FMT slurry itself. This demonstrated that BSH activity was restored (and stool TCA levels greatly reduced) to levels comparable to donors within a week of successful FMT, and was maintained at these levels at 4 weeks and 12 weeks post-FMT (p<0.05, Friedman test with Benjamini-Hochberg FDR; figure 6A,B). Similarly, BSH activity was significantly reduced in pre-FMT samples compared with either donor stool or FMT slurry (p<0.01, Mann-Whitney U test; figure 6B).

### BSH activity alone is sufficient to fully suppress TCA-mediated *C. difficile* germination in vitro

Our data demonstrated an association between successful FMT, breakdown of TCA, and restoration of gut BSH-producing microorganisms and BSH functionality. To further explore whether these changes were purely associative–or represented a true mechanistic pathway–we performed *C. difficile* batch culture germination experiments.

We prepared spent culture supernatants by incubating bacteria of interest in broth with 1% w/v TCA. After overnight incubation, cultures were centrifuged and filter-sterilised. *C. difficile* spores were incubated in sBHI broth supplemented with the spent culture supernatant. Using this set-up, *C. difficile* spores incubated with spent culture supernatants without BSH activity would have TCA available to stimulate germination and therefore grow, while *C. difficile* spores incubated with spent culture supernatants with BSH activity would not have TCA available to stimulate germination and would not grow.

**Figure 7:**
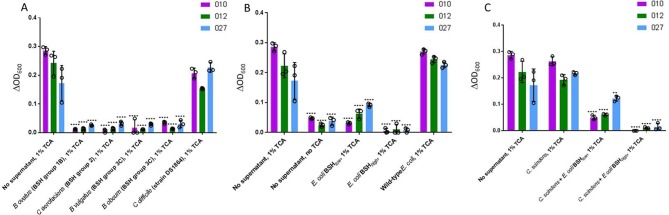
*C. difficile* batch cultures. Changes in spectrophotometer reading (ΔOD_600_) after overnight incubation of *C. difficile* spores (three ribotypes assayed: 010, 012, 027) in sBHI ±TCA in which bacterial species of interest had been cultured for 24 hours. *C. difficile* spores in sBHI supplemented with 1% TCA (‘No supernatant, 1% TCA’) was used as positive control in all cases; statistical testing shown was performed relative to this sample for the particular ribotype under assessment. (A) Batch cultures of BSH-producing microbial species found to be affected by FMT in metataxonomic analysis, and vegetative *C. difficile*. (B) Batch cultures of native *E. coli,* and two forms of *E. coli* into which *bsh* genes had been cloned *(E. coli* BSH*_low_=E. coli* expressing BSH with low deconjugation ability; *E. coli* BSH*_high_=E. coli* expressing BSH with high deconjugation activity). (C) Batch cultures of *C. scindens±*BSH expressing *E. coli* (**, p<0.01; ****, p<0.0001; analysis of variance with multiple group comparisons, Benjamini-Hochberg correction). Key: 010 is a non-toxigenic *C. difficile* ribotype, while 012 and 027 are both toxigenic ribotypes. BHI, brain heart infusion; BSH, bile salt hydrolases; TCA, taurocholic acid.

Initially, *C. difficile* spores were incubated with spent supernatant from BSH-expressing microorganisms that had been incubated with TCA. The microorganisms selected were those which had been shown to be reduced in mean proportion in the gut microbiota of pre-FMT patients in comparison to donors and/or post-FMT samples, and which collectively represented most BSH groups. For all *C. difficile* ribotypes assayed, supernatant from the broth of each of the BSH-producing microbes assayed significantly reduced *C. difficile* germination (p<0.0001) (figure 7A). As a control, we used spent supernatant from TCA-supplemented broth in which vegetative *C. difficile* had been cultured (strain DS1864); this failed to affect *C. difficile* germination.

We assessed whether BSH alone could mediate this inhibition, or if it reflected an alternative aspect of bacterial metabolism within the cultures. For subsequent batch cultures, strains of interest included wild-type *E. coli* (which lacks a *bsh* gene), or two forms of *E. coli* into which *bsh* genes had been cloned (ie, ‘*E. coli* BSH*_low_’,* containing a *bsh* gene with narrow substrate range against conjugated bile acids; and ‘*E. coli* BSH*_high_*’, containing a *bsh* gene with high glycine and taurine-deconjugating activity) (see online supplementary methods 1.7). While spent supernatant from the culture of wild-type/BSH negative *E. coli* did not affect the ability of *C. difficile* to undergo germination, that from both forms of *bsh* gene-expressing *E. coli* significantly reduced *C. difficile* germination across all ribotypes tested (p<0.0001, Student’s t-test) (figure 7B). *C. difficile* germination was significantly lower for all three ribotypes when incubated in TCA-supplemented supernatant from an *E. coli* BSH*_high_* batch culture as compared with *E. coli* BSH*_low_* (p<0.05).

*C. difficile* spores were also cultured in spent supernatant from *C. scindens* which had been incubated with TCA, either by itself or also in co-culture with BSH-expressing *E. coli. C. scindens* spent supernatant did not affect *C. difficile* germination ability by itself, but germination was significantly reduced when *C. scindens* was co-incubated with BSH-expressing *E. coli* (p<0.01) (figure 7C).

Analysis by UPLC-MS confirmed that TCA-supplemented batch culture media–which included supernatant from wild-type/BSH-negative *E. coli,* vegetative *C. difficile* and *C. scindens* culture–had high TCA:CA ratios, consistent with little/absent BSH activity within these batch cultures (see online supplementary figure 9A). Low TCA:CA ratios were noted for all other batch cultures, indicating the presence of BSH activity. All batch culture experiments in which *C. difficile* germination was suppressed were characterised by undetectable levels of DCA within supernatant (other than batch cultures containing *C. scindens* and *bsh*-expressing *E. coli,* where DCA was detectable). Sterile-filtered spent supernatant from batch cultures of BSH-producing microorganisms from groups 1, 2 and 3 was found to have BSH activity comparable to that found in healthy human stool (see online supplementary figure 9B).

### BSH limits further recurrence of CDI in a mouse model of rCDI

To investigate the hypothesis that BSH-producing bacteria limit further recurrence of disease in subjects with rCDI, we administered ~10^9^ CFU of either wild-type/BSH-negative *E. coli* or *E. coli* BSH*_high_* (n=5 in both groups) into a mouse model of rCDI shortly after completion of vancomycin (figure 1A). We used this particular protocol to aim to recapitulate the dynamics of FMT administration to patients with rCDI. Colonisation of all mice with *C. difficile* was demonstrated at 3 days post-*C. difficile* spore administration (see online supplementary figure 10A), and plate counts were used to confirm the titre of *E. coli* administered to mice (see online supplementary figure 10B). *E. coli* colonisation of the gut of mice was assessed by plate counts on selective media; we identified that on day 12 (ie, 3 days after initial *E. coli* administration), *E. coli* colonisation was at comparable, high levels in both groups of mice (mean CFU per gram of faeces of 1.49×10^9^ in mice administered *E. coli* BSH*_high_* vs 1.18×10^9^ in mice administered wild-type *E. coli*, p>0.05, Mann-Whitney U test) (figure 1B).

On day 12, *C. difficile* TVCs were significantly reduced in the mice administered *E. coli* BSH*_high_* in comparison to those administered wild-type/BSH -negative *E. coli* (figure 1C; mean CFU per gram of faeces of 6.92×10^7^ vs 2.70×10^8^, respectively; p<0.05, Mann-Whitney U test), equating to a ~70% reduction in *C. difficile* total vegetative cell counts.

## Discussion

In this study, we demonstrate for the first time that a key mechanism underlying the efficacy of FMT in treating rCDI is restoration of gut microbiota BSH functionality. Analysis of human samples illustrate that this function is restored early after FMT and maintained throughout follow-up. In addition, data from batch cultures and mouse modelling show that the BSH-mediated hydrolysis of the major *C. difficile* germinant, TCA, is sufficient to fully suppress *C. difficile* germination, and limits further recurrence of disease within the setting of rCDI. Targeted restoration of gut BSH function is a novel therapeutic approach for rCDI that avoids the risks associated with FMT.

The potential role of gut microbiota-host bile acid interactions in CDI pathogenesis has been an area of interest since initial studies in vitro established that various bile acids differentially affected the ability of *C. difficile* to undergo germination and vegetative growth.^11 12 30–33^ Furthermore, we have also recently demonstrated that successful FMT for rCDI is associated with stimulation of farnesoid X receptor (FXR) signalling,^34^ which in itself appears to impact the bile acid milieu and consequently gut microbiota of the small intestine.^35^ Subsequent studies have demonstrated that the gut of germ-free^36^ and antibiotic-treated mice^36–38^ –as well as chemostat models of CDI^8^ or humans with rCDI^39 40^–have enrichment of stool primary bile acids (particularly conjugated versions) and loss of secondary bile acids, with bile acid homeostasis being restored in patients with rCDI through FMT.^39^ Exposure of *C. difficile* spores to the bile acid milieu found in antibiotic-treated mouse caecum^37 38^ or human stool pre-FMT^41^ was sufficient to cause spore germination, while that of the non-antibiotic-treated mouse caecum^37 38^ or human stool post-FMT^41^ prevented germination and vegetative growth of *C. difficile*.

Rodent studies have demonstrated that 7-α-dehydroxylase-producing organisms (in particular, *C. scindens*) partly protected the host against the development of CDI.^42 43^ However, to date, there is not sufficient evidence to demonstrate that 7-α-dehydroxylase is responsible for the efficacy of FMT for rCDI. For example, a mouse model of CDI was successfully treated with a mixture of six bacteria, none of which are recognised to contain 7-α-dehydroxylase activity.^44^ Furthermore, *bai*CD gene abundance is not different in the stool of patients with CDI and *C. difficile*-negative patients (after correction for total bacterial load), and the *bai*CD gene is not consistently detectable in stool after successful FMT for rCDI;^45^ a study using microbial sequencing and culture demonstrated comparable results.^46^ As such, our key area of focus in this study was regarding the dynamics of BSHs in rCDI and the impact of FMT on BSH functionality. Allegretti and coauthors previously demonstrated that predicted BSH functionality was significantly reduced in the stool microbiota of patients with rCDI compared with healthy controls or those with first CDI.^14^ Our data demonstrate the restoration of BSH-producing microorganisms and associated BSH functionality post successful FMT for rCDI from the very low levels found pre-FMT back to levels similar to that of donors. This result is coupled with the loss of primary conjugated bile acids, and particularly that of the progerminant TCA.

In patients with and animal models of rCDI, few vegetative cells of *C. difficile* remain in the gut after completion of vancomycin.^26^ For further recurrence to occur, TCA-mediated germination of *C. difficile* is likely to be a key contributory factor. Our batch culture and mouse model data demonstrate that hydrolysis of TCA by BSH is sufficient to significantly limit *C. difficile* counts in rCDI. To date, no studies have been reported in mice that demonstrated restoration of 7-α-dehydroxylase activity alone could reduce *C. difficile* growth. While we have shown that degradation of TCA is the major mechanism by which restoration of BSH limits further recurrence in rCDI, an additional mechanism may be that BSH creates a larger pool of deconjugated primary bile acids, the substrate for further gut bacterial enzyme degradation and conversion of primary into secondary bile acids within the colon (see online supplementary discussion).

Given the drawbacks that currently exist regarding FMT clinically and the acknowledged need for more refined, targeted therapies, we propose that the administration of BSH-producing gut microbiota members—or the administration of purified BSH enzyme— merits further evaluation as an alternative CDI treatment strategy. Administration of a microbial community containing BSH-producing bacteria (such as those identified in this study) might be expected—like FMT—to require a single administration only, given the apparent ability of these organisms to easily colonise the gut; in contrast, to attain comparable efficacy, delivery of purified BSH enzyme is likely to require a more prolonged administration. Furthermore, while we have demonstrated proof of concept of ‘BSH therapy’ in a batch culture and mouse model setting, this would evidently merit evaluation within a clinical trial setting before it could be considered further as treatment of human patients with rCDI. It is also important to acknowledge that FMT may provide additional mechanisms of efficacy in treating rCDI (eg, potentially by competitive niche exclusion, related to the near-complete restoration of a diverse gut microbial community with high resilience^47 48^), and such benefits may be missed through the use of BSH supplementation alone.

As well as a novel treatment for CDI, BSH supplementation may be a potential novel strategy for prevention of CDI in those at high risk, for example, patients likely to require prolonged antibiotic courses. In addition, FMT has a small but appreciable failure rate, and there is currently no rational targeted biological means by which donors are selected. Assays of stool from potential donors for BSH-producing organisms and/or BSH functionality may be one such means to achieving this aim.

In conclusion, we provide evidence for the first time that microbial BSHs are a key mediator of the efficacy of FMT for rCDI. This adds to other recent data demonstrating that FMT functions by reversing a ‘metabolic dysbiosis’, including through the restoration of the short chain fatty acid valerate, which potently inhibits the growth of *C. difficile* without any apparent adverse effect on gut commensal bacteria.^8^ Furthermore, these data add further to the growing body of evidence demonstrating the central significance of gut microbiota-bile acid interactions in colonisation resistance, and demonstrate that targeted restitution of BSH may be a novel therapy for or preventative strategy against CDI that avoids the risks of antimicrobial resistance.
