# Supplementary material for: Microbial bile salt hydrolases mediate the efficacy of faecal microbiota transplant in the treatment of recurrent Clostridioides difficile infection
Source: Gut. 2019 Feb 11;68(10):1791–800. doi: 10.1136/gutjnl-2018-317842 (PMC6839797; doi:10.1136/gutjnl-2018-317842)
Supplement: Supplementary data [file gutjnl-2018-317842supp001.pdf]

Microbial bile salt hydrolases mediate the efficacy of faecal microbiota transplant in the treatment of recurrent *Clostridioides difficile* infection.

Supplementary Material:

1. Supplementary Methods:

1.1 Study participants and FMT protocols:

Clinically, participants with CDI had recurrence of diarrhoea (>3 unformed bowel movements every 24 hours) within 8 weeks of completing a prior course of treatment (with resolution of diarrhoea with anti-CDI antibiotics for prior episodes), with no clear alternate explanation for diarrhoea. CDI diagnosis was confirmed with laboratory testing, via positive ELISA for toxins A/B, with confirmation via PCR. For CDI participants, samples were collected shortly prior to FMT (whilst on suppressive doses of anti-CDI antibiotics), and again at 8-10 weeks post-successful FMT.

FMT was administered either by colonoscopy or via capsule, with 4L of polyethylene glycol bowel preparation administered to all patients on the day prior to FMT. FMT performed via colonoscopy used thawed faecal slurry which had been frozen in 10% glycerol (90% normal saline (v/v), provided by OpenBiome [1]). Capsule preparation and administration was as previously-outlined [2], and capsules contained frozen FMT with 10% glycerol as cryopreservative.

1.2 16S rRNA gene qPCR:

To quantify the biomass present in each sample we performed 16S rRNA gene qPCR using DNA extracted from stool and following a previously published protocol [3]. For each reaction, a total of 20µL was made up, consisting of the following: 1x Platinum Supermix with ROX (Life Technologies, Carlsbad, USA), 1.8 µM BactQUANT forward primer (5'-CCTACGGGAGGCAGCA-3'), 1.8 µM BactQUANT reverse primer (5'-GGACTACCGGGTATCTAATC-3'), 225nM probe ((6FAM) 5'-CAGCAGCCGCGGTA-3' (MGBNFQ)), PCR grade water (Roche, Penzberg, Germany), and 5µl DNA. For each plate we included an *E. coli* DNA (Sigma-Aldrich) standard curve, consisting of 3-300,000 copies per reaction in 10-fold serial dilutions, and a 'no template' negative control. All samples, standards, and controls were performed in triplicate. Extracted DNA samples were diluted to ensure they fell within the standard curve. The Applied Biosystems StepOnePlus Real-Time PCR System was used for amplification and real-time fluorescence detections using the following PCR cycling conditions: 50°C for 3 min, 95°C for 10 min, and 40 cycles of 95°C for 15 sec and 60°C for 1 min. We used a Mann-Whitney test to compare

16S rRNA gene copy number between donor and FMT samples, and Wilcoxon signed-rank test to compare changes between pre- and post-FMT.

1.3. Metataxonomic analysis:

The output data was analysed using the Mothur package (v1.35.1) following the MiSeq SOP Pipeline [4]. Sequence alignments were performed using the Silva bacterial database (www.arb-silva.de/), and the RDP database reference sequence files were used for sequence classification using the Wang method [5]. Operational Taxonomic unit (OTU) taxonomies (phylum to genus) were established using the RDP MultiClassifier Script. Data was resampled and normalised to the lowest read count in Mothur (11604 reads per sample), which resulted in >99.5% coverage within each sample. Where possible, species were identified from OTU data using a standard nucleotide BLAST of the 16S rRNA sequences (NCBI) with strict criteria (query cover 100% and ≥97% identity, with no other candidate species above ≥97% identity). Genus-level annotation was made where query cover was 100% and ≥94% identity. The non-metric multidimensional scaling (NMDS) plot and PERMANOVA p-values were generated using the UniFrac weighted distance matrix generated from Mothur, and analysed using the Vegan library within the R statistical package [6]. Family-level extended error bar plots were generated using the Statistical Analysis of Metagenomic Profiles (STAMP) software package using White's non-parametric *t*-test with Benjamini-Hochberg FDR [7]. The α-diversity (Shannon diversity index, H') and richness (total number of bacterial taxa observed, S_{obs}) were calculated within Mothur and statistical tests (see **Supplementary Methods 1.10**) were performed using GraphPad Prism v7.03. A *p*-value of 0.05 and a *q*-value of 0.05 was considered significant.

Changes in microbial composition were also assessed at the OTU level. Differences in mean out relative proportions > 1% were measured between donor and pre-FMT samples, and between pre-FMT and post-FMT samples, using White's non-parametric test and Benjamini-Hochberg FDR. From these data, OTUs were analysed that were enriched in donors in comparison to pre-FMT samples, and those enriched post-FMT in comparison to pre-FMT samples.

Sequencing data from this study (in fastq-format) are publicly available for download at the European Nucleotide Archive (ENA) database using study accession number PRJEB30298 (http://www.ebi.ac.uk/ena/data/view/PRJEB30298).

1.4. Prediction of bile-metabolising function of gut microbiota from metataxonomic data:

We used the inferential tool, Piphillin, to predict bile-metabolising functionality from our metaxonomic data [8,9]. This algorithm uses direct nearest-neighbour matching between 16S rRNA gene sequencing datasets and microbial genomic databases to infer the metagenomic content of samples. We used Piphillin with BioCyc version 21.0 as reference database, and applying 97% identity cut-off. Inference of gene abundance was assessed for 'choloylglycine hydrolase-RXN' (an alternative name for BSH) and for '7-alpha-hydroxysteroid-dehydrogenase-RXN'. Statistical testing between groups was performed in STAMP, using White's non-parametric *t*-test with Benjamini-Hochberg FDR.

1.5. UPLC-MS bile acid data: data pre-processing and analysis:

1.5.1. Faecal samples:

Quality control (QC) samples were prepared by pooling equal volumes of the faecal filtrates. QC samples were used as an assay performance monitor [10], and as a proxy to remove features with high variation. QC samples were also spiked with mixtures of bile acid standards (55 bile acid standards including 36 non-conjugated, 12 conjugated with taurine, seven conjugated with glycine (Steraloids, Newport, RI, USA)) and were analysed along with the stool samples to determine the chromatographic retention times of bile acids and to aid in metabolite identification.

Waters raw data files were converted to NetCDF format and data were extracted using XCMS (v1.50) package with R (v3.1.1) software. Probabilistic quotient normalisation [11] was used to correct for dilution effects and chromatographic features with coefficient of variation higher than 30% in the QC samples were excluded from further analysis. The relative intensities of the features were corrected to the dry weight of the faecal samples.

Multivariate analysis of UPLC-MS bile acid profiling data was performed on pareto-scaled data. OPLS-DA models were validated using CV-ANOVA, which provides a significance test of the null hypothesis of equal residuals between the model under validation and a randomly-fitted model which uses the same data [12]. S-plots were used to visualise the highly-influential discriminatory features, and depict the covariance and the correlation structure between the X-variables and the predictive score t[1] of the model. Features at the far ends of the plot have a very high reliability whilst having a high model influence due to their high variance in the dataset [13].

1.5.2. Batch culture supernatants:

Bile acid extraction was performed by taking 75µl of supernatant and adding 225µl of cold methanol, followed by incubation at -30°C for 2 hours; tubes were centrifuged at 9500 x *g* and 4°C for 20 min and 120µl of supernatant was loaded into vials. UPLC MS analysis was otherwise as described above.

1.6. Integration of metataxonomic and UPLC-MS bile acid data:

Regularised Canonical Correlation Analysis (rCCA) was used to correlate metataxonomic data (family-level) with UPLC-MS bile acid profiling data from the same samples using the mixOmics library within R [14,15]. This technique maximises the correlation between the two data sets X and Y. The shrinkage method was applied to determine regularisation parameters. Unit representation plots were generated using the plotIndiv function, where each sample is represented as a single point on the scatter plot, and samples were projected into XY-variate space. Correlation circle plots were generated using the plotVar function. On these plots strong correlations between variables are plotted outside of the inner circle (correlations where r>0.5). Variables are represented through their projections onto the planes defined by their respective canonical variables. Strong positive correlations are present when variables are projected in the same direction from the origin, and strong negative correlations are present when variables are projected in opposite directions. Variables present at farther distances from the origin have stronger correlations.

1.7. Bacteria used as standards for *bsh* gene qPCR and in batch cultures:

Bacteroides plebius, *Bacteroides ovatus*, *Bacteroides vulgatus, Collinsella aerofaciens* and *Blautia obeum* were previously isolated from the stool of a healthy male donor in his 20's. *Bacteroides plebius* was isolated from fastidious anaerobe agar plates (Acumedia, USA) with 5% horse blood (VWR, USA). *Bacteroides ovatus* and *Bacteroides vulgatus* were isolated from nutrient agar plates (Sigma-Aldrich, USA). *Blautia obeum* was isolated from de Man, Rogosa and Sharpe agar plates (Sigma-Aldrich). *Collinesella aerofaciens* was isolated from tryptic soy agar plates (Sigma-Aldrich).

DNA extraction was performed on the isolates using the EZNA Bacterial DNA Kit (Omega, USA) with the addition of a bead beating using the Bullet Blender Storm (speed 8 for 3 min). A ~900 bp region of the 16S gene was amplified using previously published primers[16] and DNA was sequenced at Macrogen Europe. Isolates were identified by performing a standard nucleotide BLAST of the 16S rRNA sequences (NCBI).

For qPCR, one bacterial strain from the relevant reference group was selected as a standard for each primer set (*bsh* group 1A – *Bacteroides plebius*; *bsh* group 1B – *Bacteroides ovatus*; *bsh* group 3C –

Blautia obeum; *bai*CD – *Clostridium scindens* (DSMZ 5676, Braunschweig, Germany)). Serial dilutions of each isolate were used to create a standard curve. Whilst *bsh* primers were degenerate, each primer set used was specific for an individual BSH group. The protocol used for qPCR thermocycling and gene copy number calculation was as previously-outlined [17]. For batch cultures, naturally-BSH-producing organisms that were used were: *Bacteroides ovatus* (BSH group 1B), *Collinsella aerofaciens* (group 2), *Bacteroides vulgatus* (group 3C) and *Blautia obeum* (group 3C) (two organisms were picked from group 3C given that this is a large group). The *E. coli* used that had been engineered to constitutively express *bsh* genes were: *E. coli* expressing a *bsh* gene with low activity (‘*E. coli* BSH_{low}’, with *bsh* gene cloned from *Lactobacillus salivarius,* with narrow substrate range against conjugated bile acids), and *E. coli* expressing a *bsh* gene with high activity (‘*E. coli* BSH_{high}’, with *bsh* gene cloned from *Bifidobacterium adolescentis,* containing BSH with high glycine- and taurine-deconjugating activity).

1.8. Batch cultures - *Clostridioides difficile* spore preparation and enumeration, and further methodology:

C. difficile spores were prepared using previously-described methods [18]. Specifically, *C. difficile* 010, 012 and 027 were grown anaerobically on fastidious anaerobe agar plates supplemented with 5% defibrinated horse blood (VWR, Radnor, USA) and incubated at 37°C for 7 days. The growth was removed from the plates using a sterile loop and resuspended in 1ml of sterile water. Next, 1ml of 95% ethanol was mixed with the cell suspension and was incubated for 1 hour at room temperature. The cell suspension was then centrifuged at 3000 x *g* and resuspended in 1ml sterile water. Spores were stored at -80°C until use.

Spores were enumerated by preparing serial 10-fold dilutions in PBS (Sigma-Aldrich) and plating the dilutions on Braziers Cycloserine Cefoxitin Egg Yolk agar plates (containing Braziers Cycloserine Cefoxitin Egg Yolk agar base (Lab M), cycloserine 250 mg/L (VWR), cefoxitin 8 mg/L (Sigma-Aldrich), 8% egg yolk emulsion (SLS, Nottingham, United Kingdom), 2% lysed defibrinated horse blood (VWR),

and lysozyme 5 mg/L (Sigma-Aldrich) [19]. Plates were incubated anaerobically at 37°C for 48 hours and the colonies were enumerated.

For batch cultures themselves, overnight incubation occurred at 37°C in an ElectroTek AW 400TG Anaerobic Workstation (ElectroTek, West Yorkshire, UK). Cultures were centrifuged at 20000 x *g* for 10 minutes at 4°C; supernatant from the culture was diluted 1:3 with sBHI without added TCA, and again filter sterilised (0.2μm).

1.9. Enumeration of *C. difficile* counts from mouse stool samples:

Mouse stool samples were collected into Carey-Blair medium and immediately homogenised. *Clostridioides difficile* total viable counts (TVCs) were enumerated by performing serial 10-fold dilutions of faecal supernatant in PBS and plating onto Braziers Cycloserine Cefoxitin Egg Yolk agar plates (as described earlier, with the addition of moxifloxacin 2 mg/L (VWR)) in triplicate using the method of Miles and Misra [20]. Plates were incubated anaerobically at 37°C for 2 days and colonies were enumerated.

1.10. Statistical analysis:

Multivariate UPLC-MS bile acid profiling data analysis is described in **Supplementary Methods 1.5.1.** Univariate statistics were performed using GraphPad Prism, v7.03; Mann-Whitney test was used to compare donor with pre-FMT or post-FMT, whilst Wilcoxon rank sum or Friedman test was used as appropriate to compare pre-FMT with post-FMT samples (all statistics were two-tailed tests). Correlation of metataxonomic and metabolomics data was undertaken via regularised Canonical Correlation Analysis (rCCA), using the mixOmics library within R [21].

2. Supplementary Results:

Stool from patients with rCDI demonstrated a significantly reduced α-diversity (as assessed by Shannon diversity index, $p<0.001$, Mann-Whitney, **Supplementary Figure 3A**), significantly reduced richness (S_{obs}, $p<0.0001$, **Supplementary Figure 3B**), and profoundly altered microbial community structure (as measured by NMDS, $p<0.001$, PERMANOVA, **Supplementary Figure 3C**) as compared to

healthy donors. Successful FMT was associated with restoration of all these measures to figures comparable with that of the healthy donors. All samples were inseparable by microbial community structure pre-FMT and subsequently post-FMT, regardless of whether colonoscopy or capsule administration was used (p = 0.288, PERMANOVA, **Supplementary Figure 4**).

rCDI patients had lower relative abundances of the bacterial families *Lachnospiraceae, Ruminococcaceae,* and *Bacteroidaceae,* and higher relative abundances of *Enterobacteriaceae, Lactobacillaceae* and *Veillonellaceae* compared to healthy donors (p<0.01 in all cases, Whites' non-parametric t-test with Benjamini-Hochberg correction, **Supplementary Figure 3C**). These families were all found at similar relative abundances in post-FMT and healthy donor samples. No significant difference in bacterial load (as assessed by 16S rRNA gene copy number) was observed between pre-FMT, post-FMT and donor groups (**Supplementary Figure 3D**). Analysis of metataxonomic data by specific OTU is given in **Supplementary Figure 5** and **Supplementary Table 2.**

It was noted that two species of *Lactobacilli* which produce BSH (*Lactobacillus salvarius* and *Lactobacillus plantarum*) were overall enriched in pre-FMT samples compared to donor and/ or post-FMT samples; on further analysis, this was only the case for the 4/26 patients taking probiotics prior to FMT.

The only OTU identified that could be labelled as *Clostridium scindens,* the archetypal 7-α-dehydroxylase-producing organism, was found in significantly reduced proportions between pre-FMT samples in comparison to donors (q=0.007, **Supplementary Table 2C**), but with mean proportion difference of <1%. In contrast, this OTU was not found to be significantly enriched post-FMT in comparison to pre-FMT samples (q=1.096, **Supplementary Table 2C**). However, it was noted that there was a decreased relative abundance of the genus *Clostridium* cluster XIVa (the genus most strongly associated with 7-α-dehydroxylase-producing organisms [22]) in pre-FMT samples compared to donor samples (q=0.007), and in pre-FMT samples compared to post-FMT samples (q=0.005).

3. Supplementary Discussion:

As well as degradation of TCA, an additional explanation for the association between the restitution of gut microbiota-bile acid interactions post-FMT and CDI eradication may be through the increase of secondary bile acid levels in stool. Specifically, DCA and LCA both recover post-FMT to levels commensurate with donors, which has an 'anti-*C. difficile*' effect principally through inhibition of the organism's vegetative growth [23,24]. There are at least two feasible explanations for how FMT may

contribute to this finding. One such explanation is that the restitution of gut BSH functionality post-FMT creates a larger pool of deconjugated primary bile acids, the substrate for further gut bacterial enzyme degradation and conversion of primary into secondary bile acids within the colon. An additional explanation is that FMT may also be associated with the restitution of microorganisms with 7-α-dehydroxylase activity that convert primary to secondary bile acids. We identified the enrichment in stool of unconjugated primary bile acids (chenodeoxycholic acid (CDCA) and cholic acid (CA)) and reduction in *bai*CD operon copy number pre-FMT in comparison to healthy donors, and that *bai*CD copy number/ predicted 7-α-dehydroxylase functionality was restored by FMT. It is interesting to note that *C. scindens* abundance in the gut microbiota was reduced pre-FMT in comparison with donors (albeit with <1% change in mean abundance), but there was no increase in abundance of this species post-FMT, suggesting that the increase in *bai*CD post-FMT was by yet unidentified bacteria with 7-α-dehydroxylation activity.

One drawback with interpreting our human sample data is (as is standard with most human studies of FMT and rCDI), our patients were taking vancomycin at the time of collection of pre-FMT samples, meaning that we are unable to definitively state that the restored gut BSH functionality that we observed post-FMT represents transfer of BSH-producing organisms from the donor, rather than the recovery of species in the gut microbiota of recipients that were being suppressed by vancomycin. It was for this reason that went on to perform batch culture and mouse experiments that enabled us to assess further the direct impact of BSH activity upon *C. difficile* in the rCDI setting.

There are other intuitive reasons that would support restoration of BSH-producing organisms to be a key contributor to FMT's success in the treatment of rCDI. Firstly, BSH-producing organisms have a relatively high prevalence in healthy stool, and the enzyme itself has relative insensitivity to oxygen, and robust activity over a wide pH range [25,26]. It has been demonstrated that however unrefined the preparation process of the slurry is for FMT is (i.e. regardless of ambient air, diluent or route of administration used, etc), that FMT remains highly-effective at treating rCDI. Of particular note, even a sterile faecal filtrate - prepared in a commercial blender, and not in anaerobic conditions – has been demonstrated to be of comparable efficacy to conventional FMT at treating rCDI [27]. It is more feasible that BSH (rather than 7-α-dehydroxylase) is able to reach the distal gut functionally intact after FMT (particularly in the scenario of sterile faecal filtrate, where no spores are present in the administered material, given that 7-α-dehydroxylase is produced by spore-forming *Clostridia*).

4. Supplementary Figures and Tables:

Supplementary Figure 1: Schematic of gut microbiota-bile acid interactions in humans. Taurine and glycine conjugates of the primary bile acids cholate and chenodeoxycholate are formed in the liver, and secreted through the biliary system into the small intestine. Once there, the microbially-derived enzyme bile salt hydrolase (BSH) acts to remove these taurine and glycine conjugates, reforming unconjugated cholate and chenodeoxycholate. From here, the complex, multi-step process of 7-α-dehydroxylation also occurs through microbially-derived enzymes, and converts primary to secondary bile acids (specifically, cholate is converted to deoxycholate, and chenodeoxycholate is converted to lithocholate. A range of other microbially-derived enzymes are also able to perform biotransformations upon primary bile acids [28]. Taurocholate (TCA) is the major endogenous trigger to *C. difficile* germination (with glycine as co-germinant) [23]. Cholate and deoxycholate (at high concentrations) also trigger *C. difficile* germination [2]. However (*), deoxycholate (and lithocholate) at physiological concentrations can inhibit TCA-mediated *C. difficile* germination [24,29]. Deoxycholate, lithocholate and other secondary bile acids can inhibit *C. difficile*'s vegetative growth and toxin activity [23,24,29]. Chenodeoxycholate also inhibit's *C. difficile's* germination [30]. A direct link between microbiota, bile acids and FXR signalling has been demonstrated in rodents through the use of germ-free or antibiotic-treated animals [28]. The interplay between these factors in humans remains less clear, although successful FMT for rCDI is associated with increased circulating levels of FGF19 and reduction in FGF21, consistent with increased FXR signalling post-FMT [31].

Supplementary Figure 2: Evolutionary relationships of BSH genes with their taxonomic hosts shown. The evolutionary history was inferred using the Neighbor-Joining method [32]. The optimal tree with the sum of branch length = 11.24114695 is shown. The tree is drawn to scale, with branch lengths in the same units as those of the evolutionary distances used to infer the phylogenetic tree. The evolutionary distances were computed using the Poisson correction method [33] and are in the units of the number of amino acid substitutions per site. The analysis involved 102 amino acid sequences. All positions containing gaps and missing data were eliminated. There were a total of 145 positions in the final dataset. Evolutionary analyses were conducted in MEGA7 [34].

Supplementary Figure 3: Effect of FMT for rCDI upon microbial community composition. A: Shannon diversity index for faecal samples (****, p<0.0001, Mann-Whitney test for donor vs pre-FMT, Wilcoxon rank sum test for pre-FMT vs post-FMT); B: Richness (S_{obs}, total number of bacterial taxa observed) (****, p<0.0001); C: Non-metric multidimensional scaling (NMDS) plot at family-level

(p<0.001 across all three groups, PERMANOVA); D: 16S rRNA gene copy numbers. No significant differences were found in bacterial load between groups (p>0.05 for all comparisons).

Supplementary Figure 4: NMDS plot showing different routes of FMT administration (colonoscopy vs capsule administration) for pre- and post-FMT samples. There were no significant differences between patients treated via capsule or colonoscopy pre-FMT (p = 0.288, PERMANOVA), or post-FMT (p = 0.288, PERMANOVA).

Supplementary Figure 5: OTU differences in 16S rRNA gene sequencing data in rCDI patients compared to donor or post-FMT. Extended error bar plots, with OTUs changing significantly measured by White's non-parametric test with Benjamini-Hochberg correction, using threshold of differences between mean proportions >1%. A) Donor vs pre-FMT; B) Pre-FMT vs post-FMT. Asterisks indicate OTUs changed in both comparisons.

Supplementary Figure 6: Effect of FMT for rCDI upon inferred faecal microbiota function. As established using Piphillin as inferentional tool and BioCyc database, with analysis performed in STAMP. (A) Inferred 'choloylglycine-hydrolase-RXN' functionality; (B) Inferred '7-alpha-hydroxysteroid-dehydrogenase-RXN' functionality (**, p<0.01; ****, p<0.0001, White's non-parametric t–test with Benjamini-Hochberg correction).

Supplementary Figure 7: Further UPLC-MS bile acid analysis. A: OPLS-DA scores plot, comparing donor and pre-FMT samples; B: OPLS-DA S-plot of donor vs pre-FMT data, as assessed via multivariate analysis of UPLC-MS bile acid profiling data. CA: cholic acid; CDCA: chenodeoxycholic acid; DCA: deoxycholic acid; GCA: glycocholic acid; GCDCA: glycochenodeoxycholic acid; LCA: lithocholic acid; TCA: taurocholic acid; TCDCA: taurochenodeoxycholic acid; TDCA: taurodeoxycholic acid.

Supplementary Figure 8: Univariate analysis of the effect of FMT for rCDI upon faecal profiles of specific bile acids as assessed using UPLC-MS bile acid profiling data. A: Taurocholic acid; B: Glycocholic acid; C: Chenodeoxycholic acid; D: Cholic acid; E: Taurochenodeoxycholic acid; F: Glycochenodeoxycholic acid; G: Lithocholic acid; H: Deoxycholic acid (*, p<0.05; **, p<0.01; ***, p< 0.001; ****, p<0.0001; Mann-Whitney U for donor vs pre- or post-FMT, Wilcoxon rank sum testing for pre- vs post-FMT).

Supplementary Figure 9: Additional batch culture analysis. A) Bile acid profile changes after *C. difficile* batch culture experiments, as established via UPLC-MS of media from the end of batch culture experiments. Taurocholic acid: cholic acid (TCA: CA) ratios at the end of batch culture experiments; high ratios are consistent with low/ absent BSH activity, and low ratios are consistent with high BSH activity. *C. difficile* spores in sBHI supplemented with 1% TCA ('No supernatant, 1% TCA') was used as positive control in all cases. Statistical testing shown was performed in all cases relative to 'No supernatant, 1% TCA' (****, $p<0.0001$, student t-test). B) BSH activity analysis of filter-sterilised spent supernatant from batch cultures containing microorganisms from different BSH groups. Group 1: batch culture from *Bacteroides ovatus;* Group 2: batch culture from *Collinsella aerofaciens;* Group 3: batch culture from *Bacteroides vulgatus.*

Supplementary Figure 10: Further information relating to rCDI mouse model. (A) *C. difficile* titres (expressed as CFU/g of faeces) at three days following exposure to *C. difficile* spores, as established from serial dilutions of fecal supernatant and plate counts ($p > 0.05$, Mann-Whitney U test); (B) *E. coli* titres gavaged to mice at both time points, as established from serial dilutions and plate counts ($p>0.05$, Mann-Whitney U test).

Supplementary Table 1: Clinical characteristics of FMT recipients.

Supplementary Table 2: Changes in mean proportions of OTUs after FMT for rCDI: A) OTUs enriched in donors compared to pre-FMT; B) OTUs enriched post-FMT compared to pre-FMT; C) Changes in the OTU corresponding to *Clostridium scindens,* the archetypal 7-α-dehydroxylase-producing bacterium. Annotation of species was performed after running OTU sequencing through Microbial BLAST. Where criteria were not met for annotation of genus or species, annotations as assigned by Mothur were used instead (these are the OTUs where no query cover or identity figure is quoted). OTUs highlighted in blue: microbes with *bsh* genes.

Supplementary Table 3: Model characteristics for OPLS-DA models. Validation of the models is expressed with p values derived from CV-ANOVA.

5. References:

1 OpenBiome. https://www.openbiome.org/ (accessed 1 Feb 2018).

2 Kao D, Roach B, Silva M, *et al.* Effect of Oral Capsule– vs Colonoscopy-Delivered Fecal Microbiota Transplantation on Recurrent *Clostridium difficile* Infection. *JAMA* 2017;**318**:1985.

doi:10.1001/jama.2017.17077

3 Liu CM, Aziz M, Kachur S, *et al.* BactQuant: An enhanced broad-coverage bacterial quantitative real-time PCR assay. *BMC Microbiol* 2012;**12**:56. doi:10.1186/1471-2180-12-56

4 Kozich JJ, Westcott SL, Baxter NT, *et al.* Development of a dual-index sequencing strategy and curation pipeline for analyzing amplicon sequence data on the MiSeq Illumina sequencing platform. *Appl Environ Microbiol* 2013;**79**:5112–20. doi:10.1128/AEM.01043-13

5 Wang Q, Garrity GM, Tiedje JM, *et al.* Naive Bayesian Classifier for Rapid Assignment of rRNA Sequences into the New Bacterial Taxonomy. *Appl Environ Microbiol* 2007;**73**:5261–7. doi:10.1128/AEM.00062-07

6 R: The R Project for Statistical Computing. https://www.r-project.org/ (accessed 27 Nov 2017).

7 Parks DH, Tyson GW, Hugenholtz P, *et al.* STAMP: statistical analysis of taxonomic and functional profiles. *Bioinformatics* 2014;**30**:3123–4. doi:10.1093/bioinformatics/btu494

8 Second Genome Therapeutics. Piphillin. http://secondgenome.com/solutions/resources/data-analysis-tools/piphillin/ (accessed 14 Feb 2018).

9 Iwai S, Weinmaier T, Schmidt BL, *et al.* Piphillin: Improved Prediction of Metagenomic Content by Direct Inference from Human Microbiomes. *PLoS One* 2016;**11**:e0166104. doi:10.1371/journal.pone.0166104

10 Sangster T, Major H, Plumb R, *et al.* A pragmatic and readily implemented quality control strategy for HPLC-MS and GC-MS-based metabonomic analysis. *Analyst* 2006;**131**:1075. doi:10.1039/b604498k

11 Veselkov KA, Vingara LK, Masson P, *et al.* Optimized Preprocessing of Ultra-Performance Liquid Chromatography/Mass Spectrometry Urinary Metabolic Profiles for Improved Information Recovery. *Anal Chem* 2011;**83**:5864–72. doi:10.1021/ac201065j

12 Eriksson L, Trygg J, Wold S. CV-ANOVA for significance testing of PLS and OPLS® models. *J Chemom* 2008;**22**:594–600. doi:10.1002/cem.1187

13 Wiklund S, Johansson E, Sjöström L, *et al.* Visualization of GC/TOF-MS-Based Metabolomics Data for Identification of Biochemically Interesting Compounds Using OPLS Class Models. *Anal Chem* 2008;**80**:115–22. doi:10.1021/ac0713510

14 Le Cao, Kim-Anh, Rohart F, Gonzalez I DS. CRAN - Package mixOmics. https://cran.r-project.org/web/packages/mixOmics/index.html (accessed 24 Jan 2018).

15 Gonzalez I, Déjean S, Martin P, *et al.* **CCA** : An *R* Package to Extend Canonical Correlation Analysis. *J Stat Softw* 2008;**23**:1–14. doi:10.18637/jss.v023.i12

16 Ben-Dov E, Shapiro OH, Siboni N, *et al.* Advantage of using inosine at the 3' termini of 16S rRNA gene universal primers for the study of microbial diversity. *Appl Environ Microbiol* 2006;**72**:6902–6. doi:10.1128/AEM.00849-06

17 Mullish BH, Pechlivanis A, Barker GF, *et al.* Functional microbiomics: Evaluation of gut microbiota-bile acid metabolism interactions in health and disease. Methods. 2018. doi:10.1016/j.ymeth.2018.04.028

18 Freeman J, O 'neill FJ, Wilcox MH. Effects of cefotaxime and desacetylcefotaxime upon Clostridium difficile proliferation and toxin production in a triple-stage chemostat model of the human gut. *J Antimicrob Chemother* 2003;**52**:96–102. doi:10.1093/jac/dkg267

19 Crowther GS, Chilton CH, Todhunter SL, *et al.* Comparison of planktonic and biofilm-associated communities of Clostridium difficile and indigenous gut microbiota in a triple-stage chemostat gut model. *J Antimicrob Chemother* 2014;**69**:2137–47. doi:10.1093/jac/dku116

20 Miles AA, Misra SS, Irwin JO. The estimation of the bactericidal power of the blood. *J Hyg (Lond)* 1938;**38**:732–49.http://www.ncbi.nlm.nih.gov/pubmed/20475467 (accessed 5 Oct 2018).

21 CRAN - Package mixOmics. https://cran.r-project.org/web/packages/mixOmics/index.html (accessed 3 Jan 2018).

22 Ridlon JM, Kang D-J, Hylemon PB. Bile salt biotransformations by human intestinal bacteria. *J Lipid Res* 2006;**47**:241–59. doi:10.1194/jlr.R500013-JLR200

23 Sorg JA, Sonenshein AL. Bile Salts and Glycine as Cogerminants for Clostridium difficile Spores. *J Bacteriol* 2008;**190**:2505–12. doi:10.1128/JB.01765-07

24 Thanissery R, Winston JA, Theriot CM. Inhibition of spore germination, growth, and toxin activity of clinically relevant C. difficile strains by gut microbiota derived secondary bile acids. *Anaerobe* 2017;**45**:86–100. doi:10.1016/j.anaerobe.2017.03.004

25 Thomas LA, King A, French GL, *et al.* Cholylglycine hydrolase and 7α-dehydroxylase optimum assay conditions in vitro and caecal enzyme activities ex vivo. *Clin Chim Acta* 1997;**268**:61–72. doi:10.1016/S0009-8981(97)00169-1

26 Begley M, Hill C, Gahan CGM. Bile salt hydrolase activity in probiotics. *Appl Environ Microbiol* 2006;**72**:1729–38. doi:10.1128/AEM.72.3.1729-1738.2006

27 Ott SJ, Waetzig GH, Rehman A, *et al.* Efficacy of Sterile Fecal Filtrate Transfer for Treating Patients With Clostridium difficile Infection. *Gastroenterology* 2017;**152**:799–811.e7. doi:10.1053/j.gastro.2016.11.010

28 Wahlström A, Sayin SI, Marschall HU, *et al.* Intestinal Crosstalk between Bile Acids and

Microbiota and Its Impact on Host Metabolism. Cell Metab. 2016;**24**:41–50. doi:10.1016/j.cmet.2016.05.005

29 Theriot CM, Bowman AA, Young VB. Antibiotic-Induced Alterations of the Gut Microbiota Alter Secondary Bile Acid Production and Allow for Clostridium difficile Spore Germination and Outgrowth in the Large Intestine. doi:10.1128/mSphere.00045-15

30 Sorg JA, Sonenshein AL. Chenodeoxycholate Is an Inhibitor of *Clostridium difficile* Spore Germination. *J Bacteriol* 2009;**191**:1115–7. doi:10.1128/JB.01260-08

31 Monaghan T, Mullish BH, Patterson J, *et al.* Effective fecal microbiota transplantation for recurrent Clostridioides difficile infection in humans is associated with increased signalling in the bile acid-farnesoid X receptor-fibroblast growth factor pathway. *Gut Microbes* 2018;:1–7. doi:10.1080/19490976.2018.1506667

32 Zuckerlandl E, Pauling L. The neighbor-joining method: A new method for reconstructing phylogenetic trees. *Mol Biol Evol* 1965;**4**:406–25.

33 Bryson V, Vogel HJ (Henry J, Rutgers University. Institute of Microbiology. *Evolving genes and proteins : a symposium held at the Institute of Microbiology of Rutgers, the State University, with support from the National Science Foundation*. Academic Press 1965. https://www.sciencedirect.com/science/book/9781483227344#ancpart3 (accessed 28 Feb 2018).

34 Kumar S, Stecher G, Tamura K. MEGA7: Molecular Evolutionary Genetics Analysis Version 7.0 for Bigger Datasets. *Mol Biol Evol* 2016;**33**:1870–4. doi:10.1093/molbev/msw054

Supplementary Figure 1:

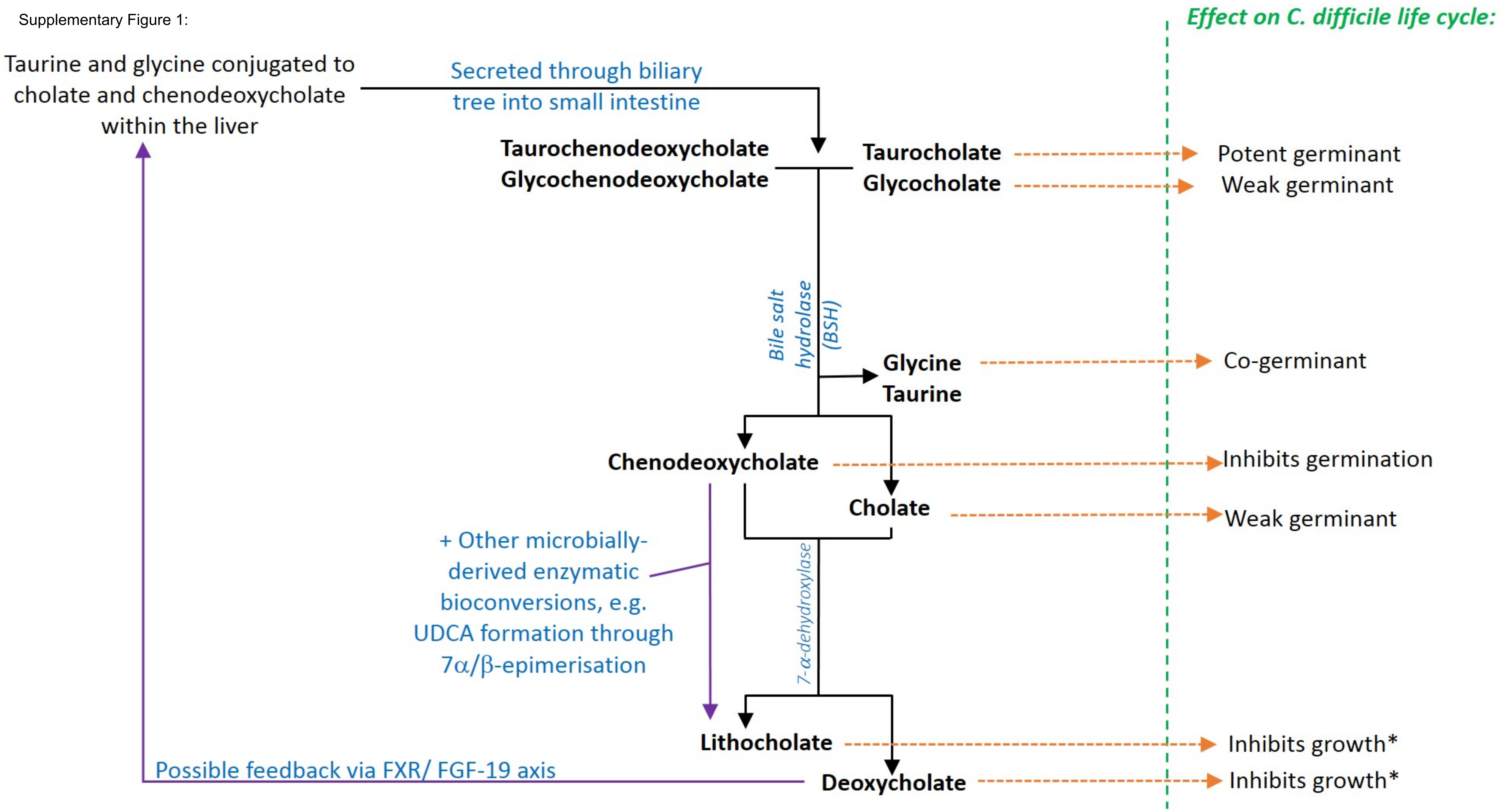

Supplementary Figure 2:

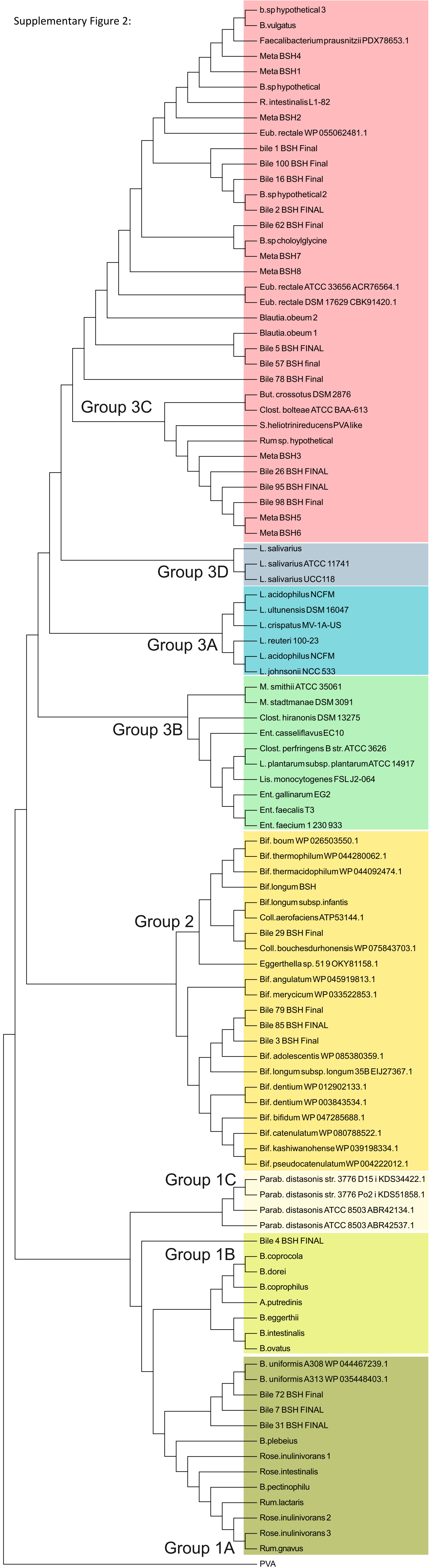

Supplementary Figure 3:

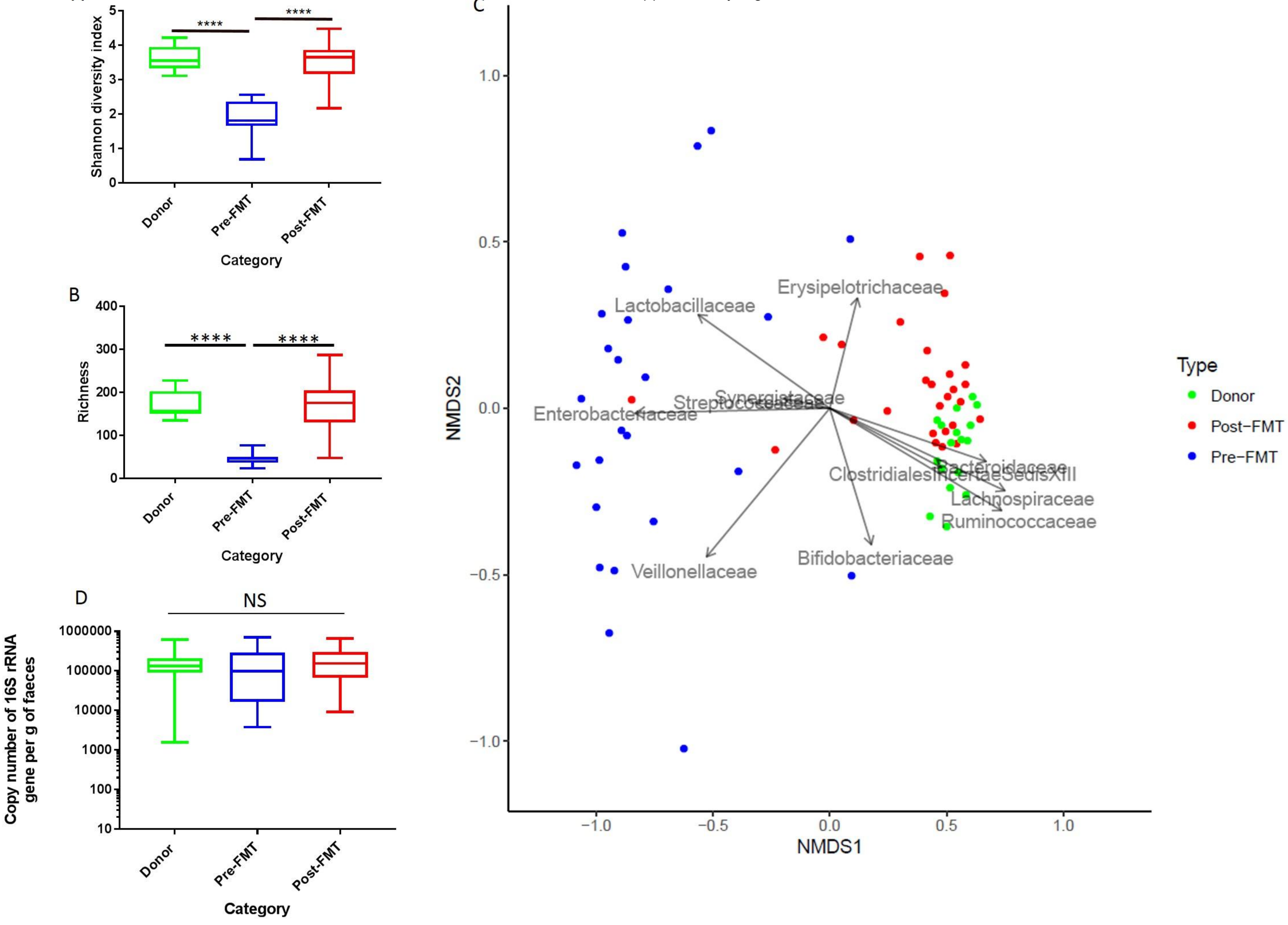

Supplementary Figure 4:

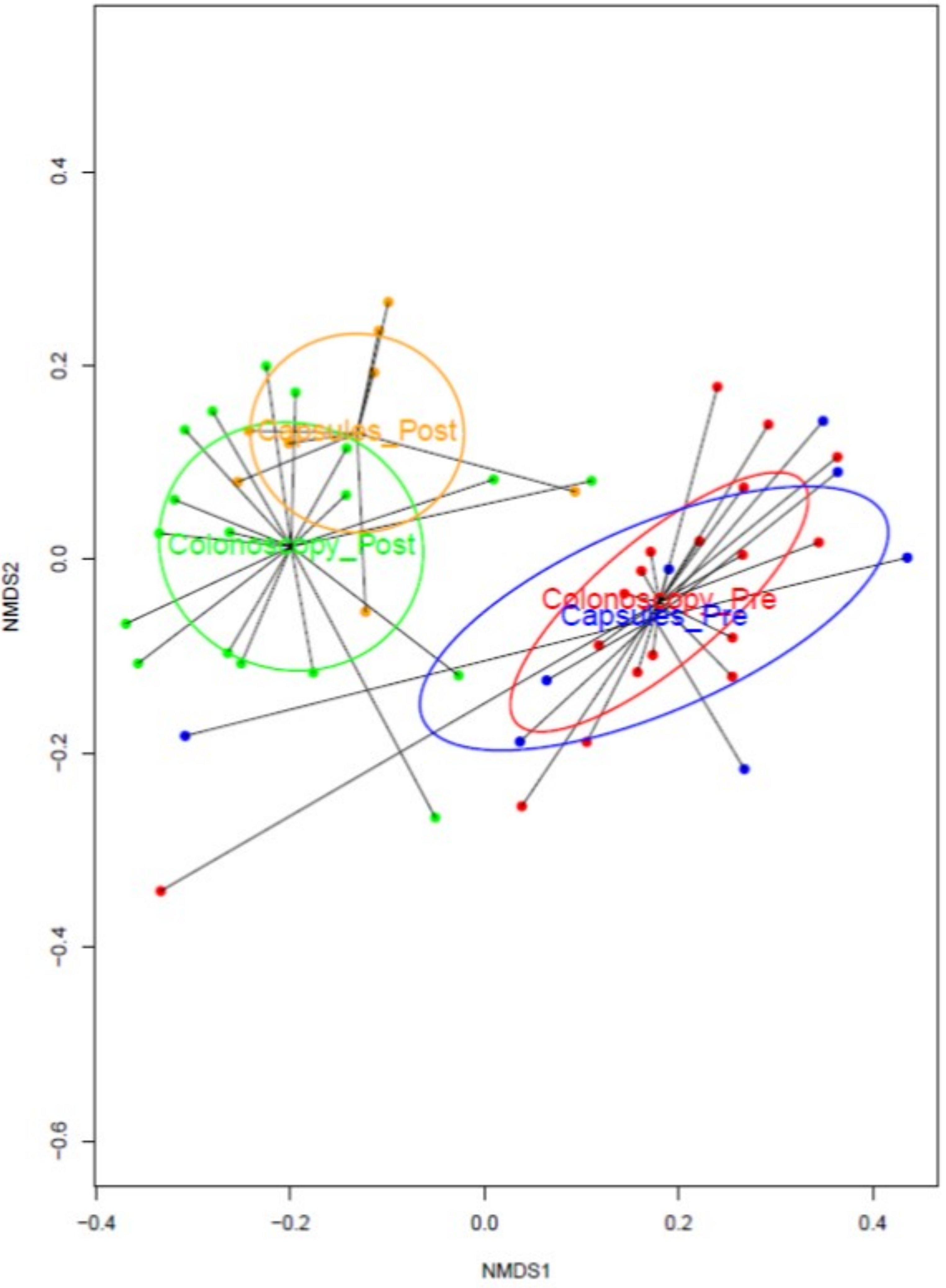

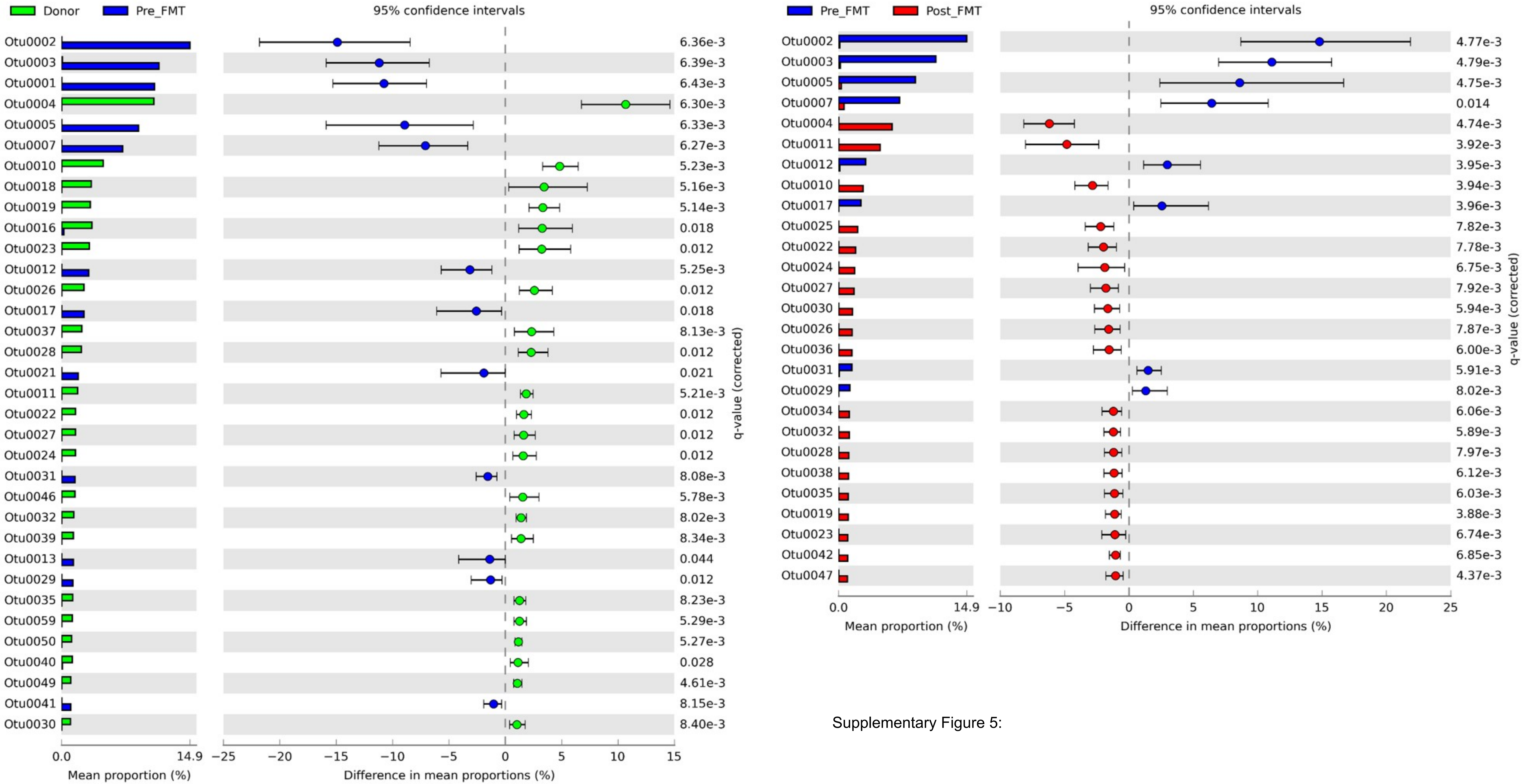

Supplementary Figure 5:

Supplementary Figure 6:

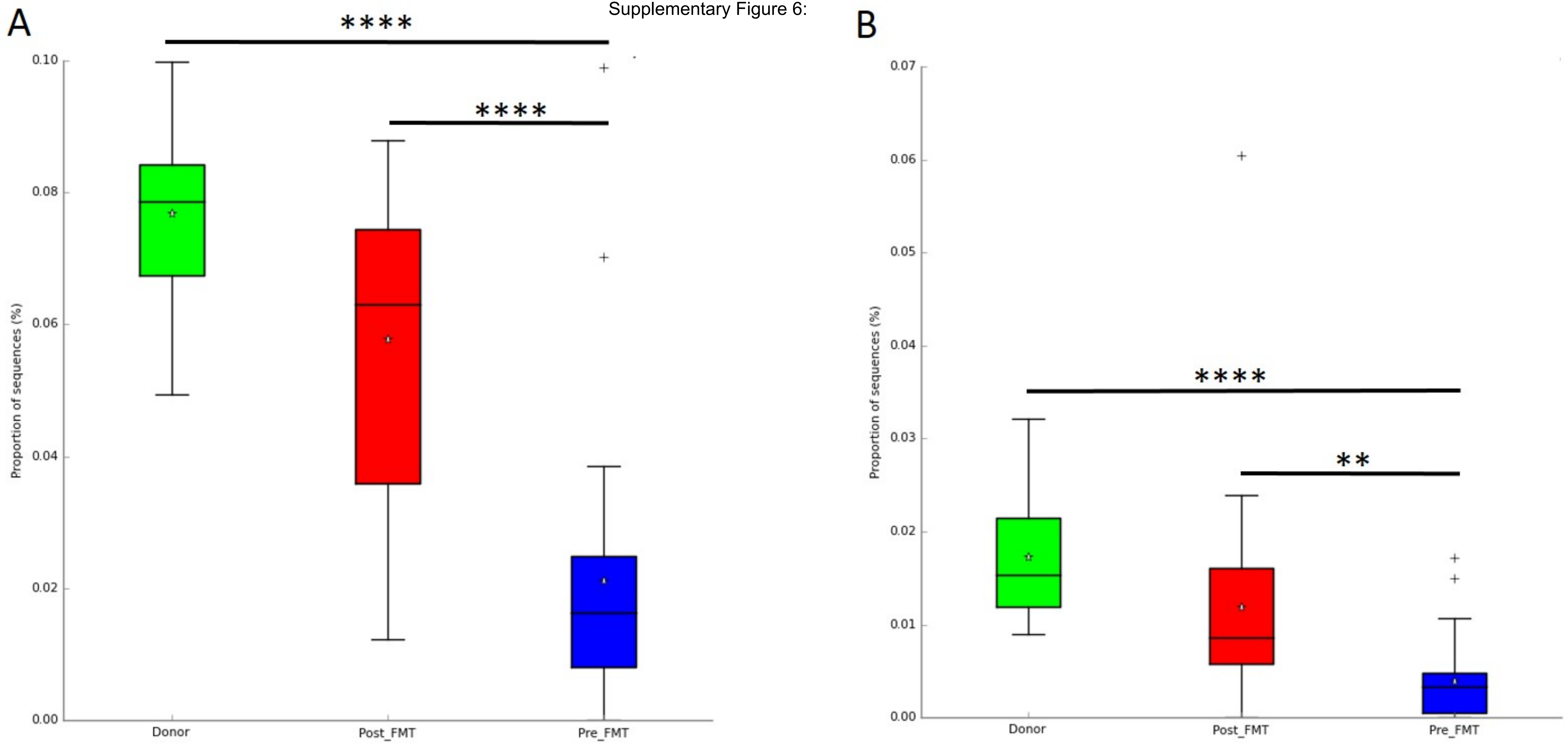

Supplementary Figure 7:

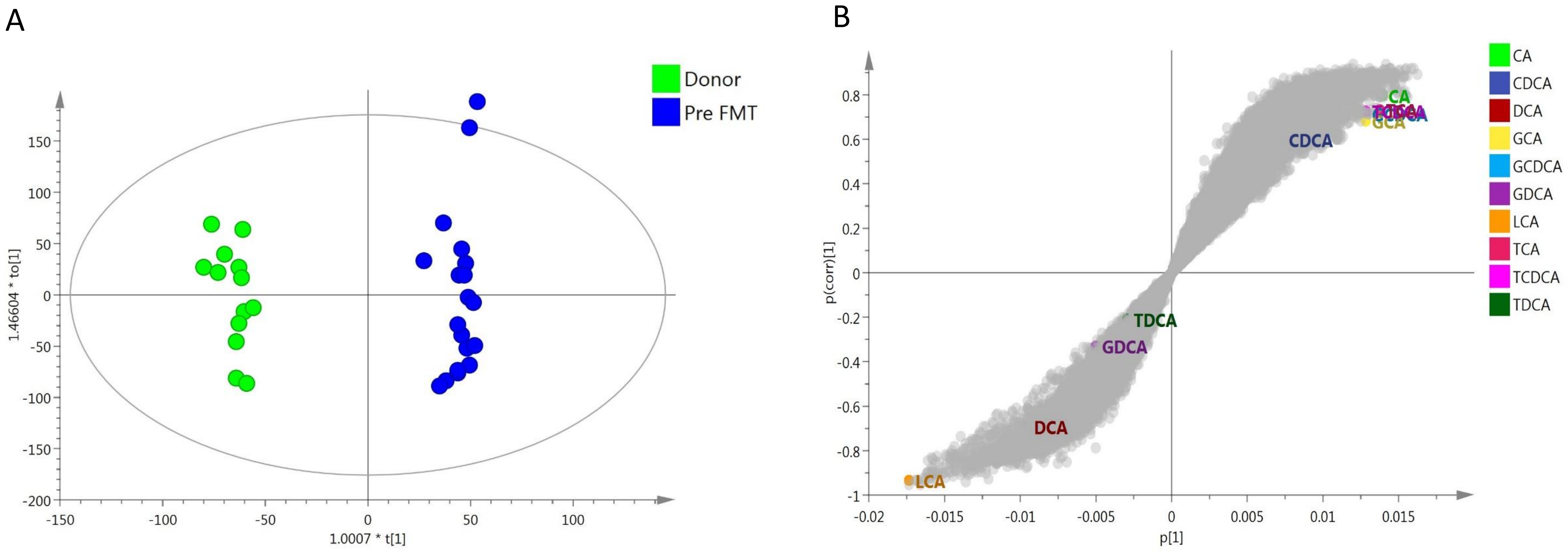

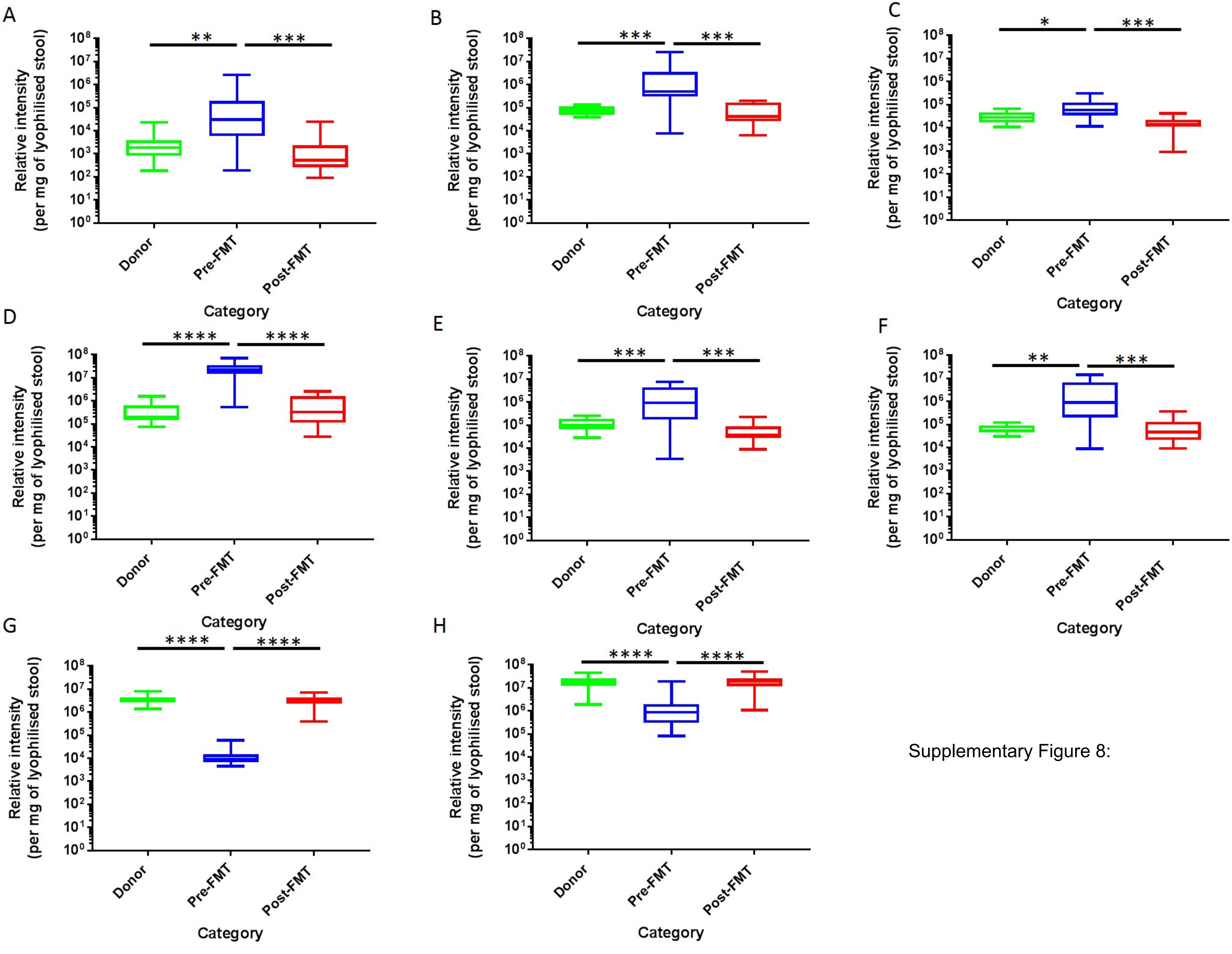

Supplementary Figure 8:

Supplementary Figure 9:

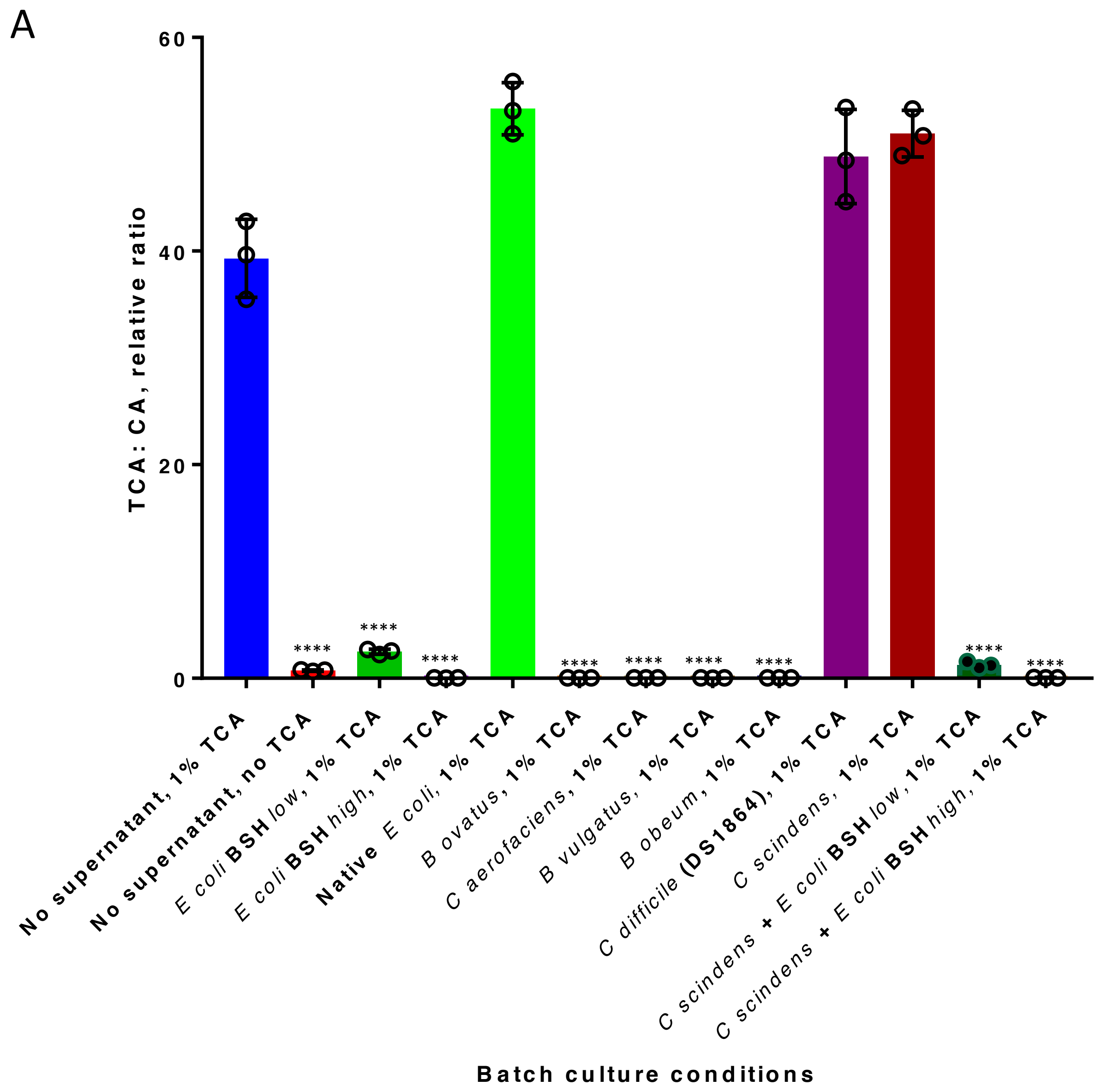

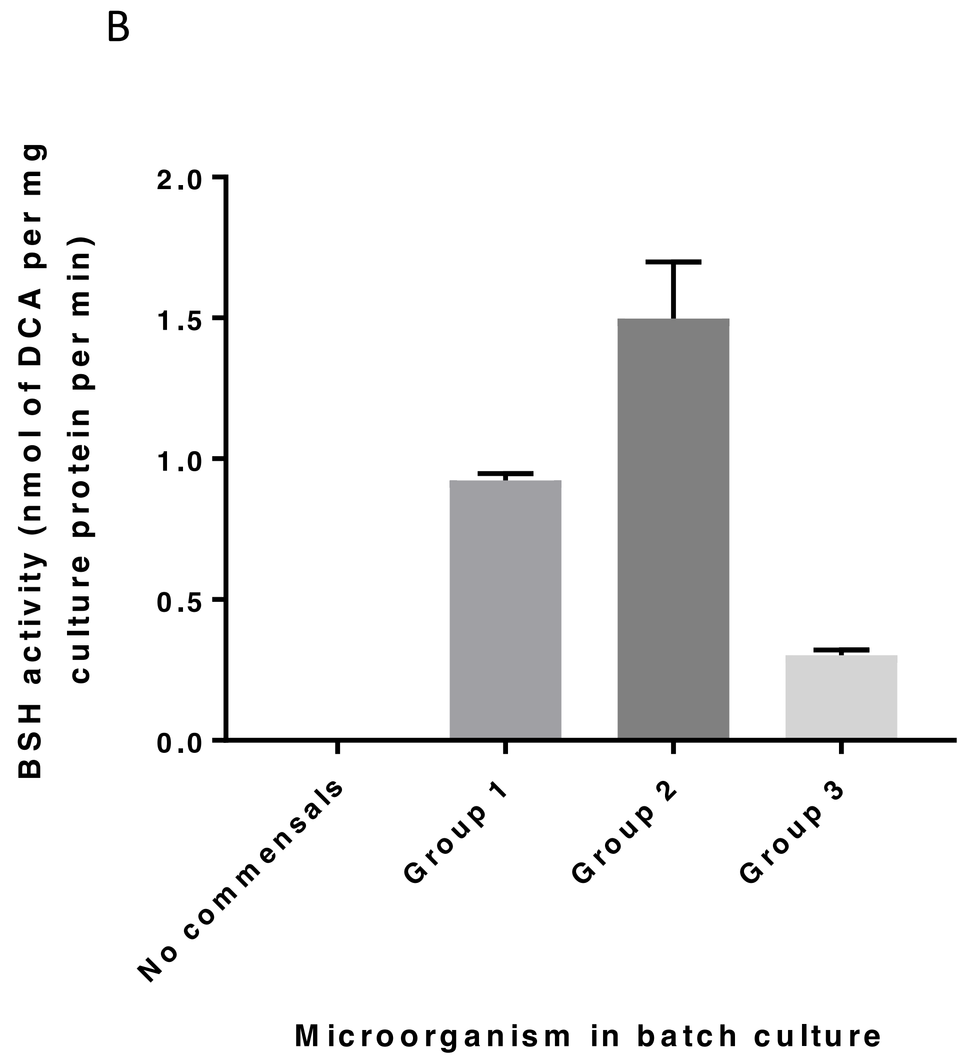

Supplementary Figure 10:

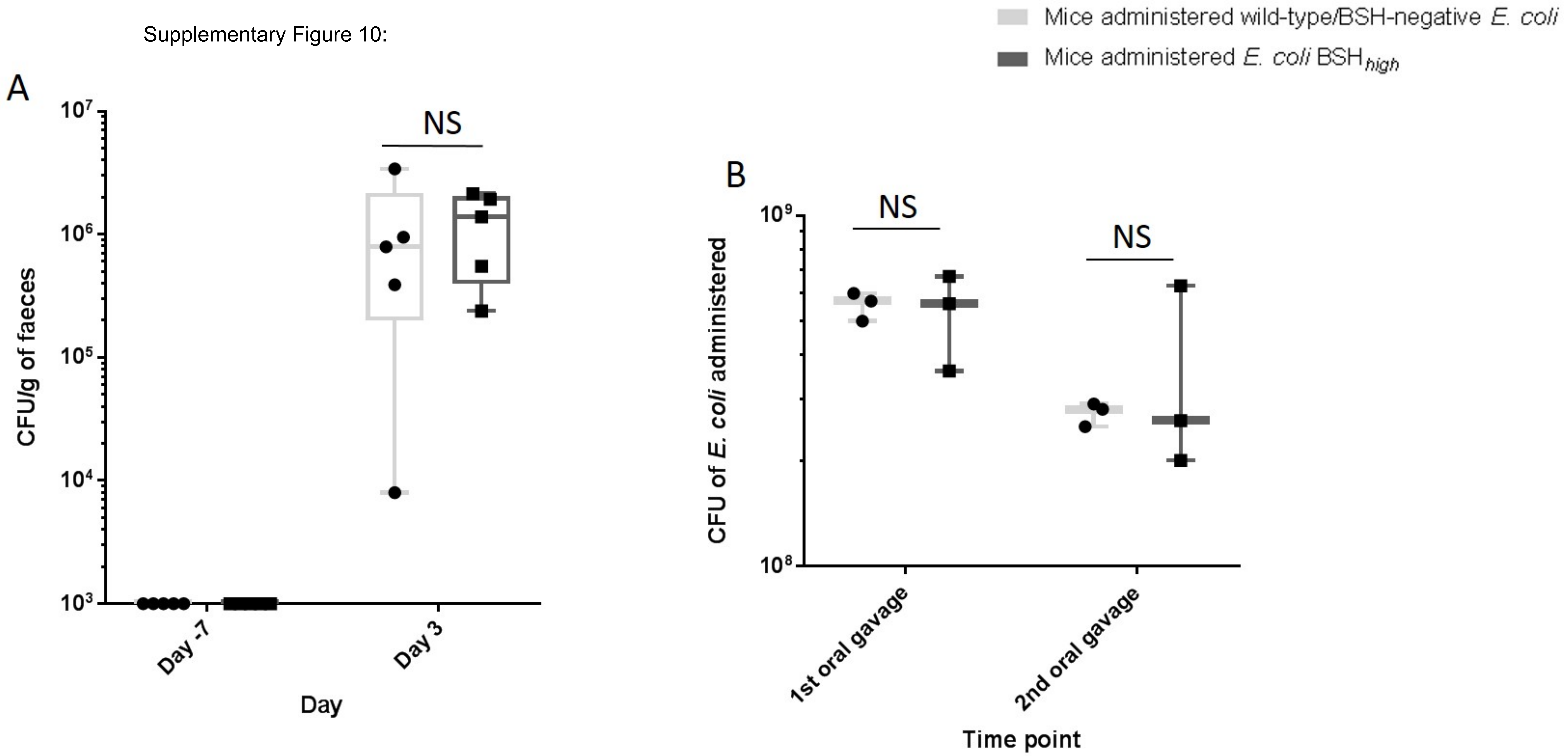

Supplementary Table 1:

Characteristic	Value
Age (years) (mean +/- SD)	66 +/- 13
Sex	65% female (17/26)
BMI pre-FMT (kg/m^2)	25.01 +/- 7
Median recurrences	3 (range: 1 - 5)
Median prior CDI-related hospitalisations	1 (range: 1-3)
Reported antibiotic use prior to CDI	88% (23/26)
Reported previous failed vancomycin taper	58% (15/26)
Route of administration	31% capsulised (8/26), 69% colonoscopy (18/26)
PPI use	42% (11/26)
Statin use	12% (3/26)

Supplementary Table 2:

A

OTUs enriched in donors vs pre-FMT:				
OTU	Identity	Query cover (%)	Identity (%)	q value for difference in mean proportions (corrected)
Otu0004	*Bacteroides vulgatus*	100	100	0.00630
Otu0010	*Eubacterium rectale*	100	100	0.00523
Otu0018	Unclassified *Bacteroides*	100	96	0.00516
Otu0019	*Faecalibacterium prausnitzii*	100	99	0.00514
Otu0016	*Dialister invisus*	100	100	0.018
Otu0023	Unclassified *Bacteroides*	-	-	0.012
Otu0026	*Bifidobacterium adolescentis*	100	100	0.012
	Bifidobacterium dentium	100	97	
Otu0037	*Bacteroides caccae*	100	99	0.00813
Otu0028	*Bacteroides ovatus*	100	99	0.012
Otu0011	*Bacteroides uniformis*	100	99	0.00521
Otu0022	*Bacterium LF-3*	100	100	0.012
Otu0027	*Collinsella aerofaciens*	100	99	0.012
Otu0024	Unclassified *Bacteroides*	100	94	0.012
Otu0046	*Bacteroides stercoris*	100	99	0.00578
Otu0032	*Anaerostipes hadrus*	100	100	0.00802
Otu0039	Unclassified *Ruminococcaceae*	-	-	0.00834
Otu0035	Unclassified *Oscillibacter*	-	-	0.00832
Otu0059	*Faecalibacterium prausnitzii*	100	98	0.00529
Otu0050	*Fusicatenibacter saccharivorans*	100	99	0.00527
Otu0040	*Bifidobacterium longum*	100	100	0.028
	Bifidobacterium sanguini	100	97	
Otu0049	Unclassified *Lachnospiraceae*	-	-	0.00461
Otu0030	*Barnesiella intestinihominis*	100	99	0.00840

B

OTUs enriched post-FMT compared to pre-FMT:				
OTU	Identity	Query cover (%)	Identity (%)	q value for difference in mean proportions (corrected)
Otu0004	*Bacteroides vulgatus*	100	100	0.00474
Otu0011	*Bacteroides uniformis*	100	99	0.00392
Otu0010	*Eubacterium rectale*	100	100	0.00394
Otu0025	*Parabacteroides johnsonii*	100	98	0.00782
Otu0022	*Bacterium LF-3*	100	100	0.00778
Otu0024	Unclassified *Bacteroides*	100	94	0.00675
Otu0027	*Collinsella aerofaciens*	100	99	0.00792
Otu0030	*Barnesiella intestinihominis*	100	99	0.00594
Otu0026	*Bifidobacterium adolescentis*	100	100	0.00787
	Bifidobacterium dentium	100	97	
Otu0036	*Bacteroides cellulosilyticus*	100	98	0.00600
Otu0034	*Blautia obeum*	100	97	0.00606
Otu0032	*Anaerostipes hadrus*	100	100	0.00589
Otu0028	*Bacteroides ovatus*	100	99	0.00797
Otu0038	*Bacteroides ovatus*	100	97	0.00612
Otu0035	Unclassified *Oscillibacter*	-	-	0.00603
Otu0019	*Faecalibacterium prausnitzii*	100	99	0.00388
Otu0023	Unclassified *Bacteroides*	-	-	0.00674
Otu0042	*Blautia obeum*	100	98	0.00685
Otu0047	*Parabacteroides distasonis*	100	98	0.00437

C

OTUs enriched in donors vs pre-FMT:				
OTU	Identity	Query cover (%)	Identity (%)	q value for difference in mean proportions (corrected)
Otu0212	*Clostridium scindens*	100	99	0.00663
**OTUs enriched post-FMT compared to pre-FMT:**				
OTU	Identity	Query cover (%)	Identity (%)	q value for difference in mean proportions (corrected)
Otu0212	*Clostridium scindens*	100	99	1.096

Supplementary Table 3:

**Multi-variate model**	**R^2X**	**Q^2**	***p* value**
Donor vs pre-FMT	0.533	0.933	1.85×10^{-13}
Pre- vs post-FMT	0.437	0.839	5.24×10^{-13}
